# A powerful long metabarcoding method for the determination of complex diets from faecal analysis of the European pond turtle (*Emys orbicularis*, L. 1758)

**DOI:** 10.1111/1755-0998.13277

**Published:** 2020-11-04

**Authors:** Charlotte Ducotterd, Julien Crovadore, François Lefort, Jean‐François Rubin, Sylvain Ursenbacher

**Affiliations:** ^1^ Department of Ecology and Evolution University of Lausanne Lausanne Switzerland; ^2^ Centre Emys Association de Protection et Récupération des Tortues Chavornay Switzerland; ^3^ La Maison de la Rivière Tolochenaz Switzerland; ^4^ HEPIA HES‐SO University of Applied Sciences and Arts Western Switzerland Jussy Switzerland; ^5^ Department of Environmental Sciences Section of Conservation Biology University of Basel Basel Switzerland; ^6^ info fauna – Centre Suisse de Cartographie de la Faune (CSCF) and Centre de coordination pour les reptiles et les amphibiens de Suisse (Karch) Neuchâtel Switzerland

**Keywords:** de novo assembly diet analysis, European pond turtle, faecal analysis, long metabarcoding

## Abstract

High‐throughput sequencing has become an accurate method for the identification of species present in soil, water, faeces, gut or stomach contents. However, information at the species level is limited due to the choice of short barcodes and based on the idea that DNA is too degraded to allow longer sequences to be amplified. We have therefore developed a long DNA metabarcoding method based on the sequencing of short reads followed by de novo assembly, which can precisely identify the taxonomic groups of organisms associated with complex diets, such as omnivorous individuals. The procedure includes 11 different primer pairs targeting the COI gene, the large subunit of the ribulose‐1,5‐bisphosphate carboxylase gene, the maturase K gene, the 28S rRNA and the *trnL‐trnF* chloroplastic region. We validated this approach using 32 faeces samples from an omnivorous reptile, the European pond turtle (*Emys orbicularis*, L. 1758). This metabarcoding approach was assessed using controlled experiments including mock communities and faecal samples from captive feeding trials. The method allowed us to accurately identify prey DNA present in the diet of the European pond turtles to the species level in most of the cases (82.4%), based on the amplicon lengths of multiple markers (168–1,379 bp, average 546 bp), and produced by de novo assembly. The proposed approach can be adapted to analyse various diets, in numerous conservation and ecological applications. It is consequently appropriate for detecting fine dietary variations among individuals, populations and species as well as for the identification of rare food items.

## 1 INTRODUCTION

Molecular technologies, such as high‐throughput amplicon sequencing (HTS), have become a method of choice to accurately and rapidly characterize complex, multispecies, ecological communities. This approach has the potential to greatly improve the accuracy of diet analysis from faecal samples or stomach contents (Alberdi et al., 2018). HTS has been used to assess the diet composition of a wide taxonomic range of animals. Animals whose diets have been successfully investigated have included mammals (Buglione et al., 2018; De Barba et al., 2014; Esnaola et al., 2018; Robeson et al., 2017), birds (Crisol‐Martinìez et al., 2016; Han & Oh, 2018), reptiles (Caut et al., 2019; Kartzinel & Pringle, 2015; Koizumi et al., 2017), fish (Barbato et al., 2019; Harms‐Tuohy et al., 2016; Riccioni et al., 2018) and arthropods (Kamenova et al., 2018; Kennedy et al., 2020; Krehenwinkel et al., 2016). For most species, faecal samples, in contrast to stomach contents, can easily be obtained, with a minimal level of interaction and harm inflicted on the studied animal. Using faecal samples is therefore a noninvasive and attractive approach to study dietary patterns (Pompanon et al., 2012; Valentini et al., 2009). This is especially true for endangered species or species whose feeding patterns are difficult to observe in the wild, such as aquatic or nocturnal species (Baamrane et al., 2012; Hibert et al., 2013). Following sampling, laboratory procedures involve total DNA extraction from faecal samples, PCR amplification with either a universal or a specific set of primers corresponding to one or more barcode loci, preparation of DNA libraries and DNA sequencing ultimately terminated by data processing via bioinformatics pipelines (Laudadio et al., 2019).

Previous studies have revealed the difficulty associated with determining operational taxonomic units (OTUs) at the species level, such as has been reported in diet (*Ursus arctos*: De Barba et al., 2014; *Lepus corsicanus*: Buglione et al., 2018) or environmental DNA (eDNA) studies (Lacoursière‐Roussel et al., 2018; Lim et al., 2016; Ruppert et al., 2019). Furthermore, obtaining sufficient amplicon length to determine sample identities to the species level can be challenging (De Barba et al., 2014; Deagle et al., 2010). This difficulty is primarily due to the fact that prey DNA in faecal samples is degraded (Deagle et al., 2006). Consequently, primers were selected to target only short and variable prey DNA fragments present in the diet. Long metabarcoding has facilitated the production of long sequencing reads (Godwin et al., 2016). The increased marker length allows a higher taxonomic resolution, with long markers increasing the ability to distinguish closely related species (Singer et al., 2016). Regarding vertebrates and invertebrates, the mitochondrial cytochrome oxidase subunit 1 (COI) is the most frequently used barcode locus, with the most diversified and complete reference database (Jusino et al., 2018). The careful selection and design of amplification primers are essential, as well as the evaluation of primers from close and distant species, using as many DNA sources as possible, in order to characterize primer specificity and/or universality.

Together with primer selection, the parameters of bioinformatic pipelines have an important impact on the identification of OTUs. Indeed, after sequencing, to achieve in‐depth analysis of DNA present in the studied sample, raw sequence data alone are not sufficient (van der Walt et al., 2017). Furthermore, unassembled raw metagenomic sequence data are fragmented, contain errors and/or are affected by unequal sequencing depths (Nagarajan & Pop, 2013), hindering the accuracy when sorting DNA sequences (Nurk et al., 2017). Thus, to accurately analyse metagenomes, larger contiguous segments named contigs can be assembled from raw sequence data (Anantharaman et al., 2016). For this reason, multiple metagenome bioinformatic pipelines have been developed to assemble raw sequences by simply merging paired‐end reads or by de novo assembly (van der Walt et al., 2017).

Using a metagenome assembler producing high‐quantity long contigs (>1,000 bp) will allow for more accurate determination of organisms to the species level using DNA sequences present in the sample (van der Walt et al., 2017). Additionally, control and validation methods are needed for parametrizing bioinformatics pipelines. This can be achieved by creating and sequencing mock communities, which are references for DNA databases and are used as positive controls for HTS (Jusino et al., 2018). Moreover, a captive feeding trial can be conducted to evaluate the applicability of the method and to test whether prey DNA can be reliably detected in faecal samples (Deagle et al., 2005; Nakahara et al., 2015).

Here, we describe a study of the complex diet of the European pond turtle, using, for the first time in a dietary study, a new long DNA metabarcoding approach. The procedure includes 11 different primers pairs that target a region of the COI gene, the large subunit of the ribulose‐1,5‐bisphosphate carboxylase gene (*rbcL*), the maturase K gene (*matK*), the 28S rRNA and the *trnL‐trnF* chloroplastic region (the proposed combination of primers covers plants, invertebrates and vertebrates). This will result in the amplification of fragments between 350 and 1,400 bp in length. After sequencing, raw metagenome sequence data are first analysed with the open bioinformatics pipeline, metaspades version 3.9.0 (Nurk et al., 2017; http://cab.spbu.ru/software/spades), which, to our knowledge, is being used for the first time in diet studies using metagenomic approaches, and considered nowadays as the most recommended metagenomic data assembler for high‐complexity metagenomes (Forouzan et al., 2018). This new DNA metabarcoding approach is based on the use of multiple primers in order to maximize coverage of species groups and can accurately be used to identify taxa to the species level for plants, vertebrates and invertebrates present in faeces collected in the field. Finally, the accuracy of the method was also validated by using faeces obtained from captive European pond turtles fed using known diets.

## 2 MATERIALS AND METHODS

### 2.1 General approach for omnivorous diet analysis

**FIGURE 1:**
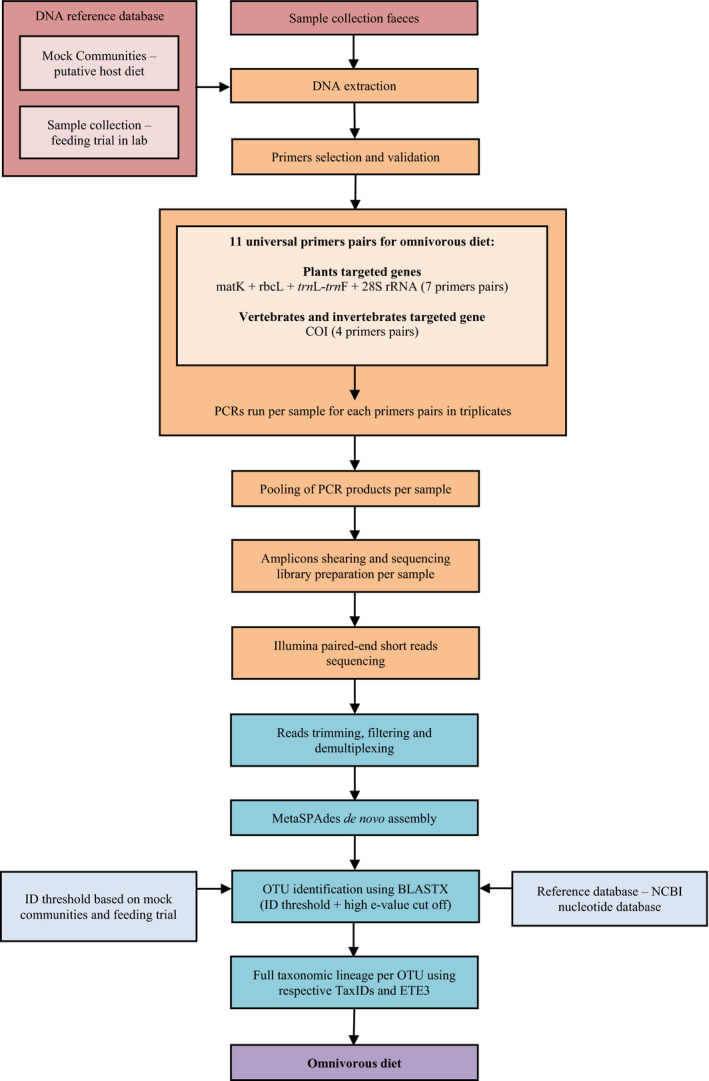
Flowchart summarizing the experimental design for the long DNA metabarcoding approach for omnivorous diet analysis [Colour figure can be viewed at wileyonlinelibrary.com]

We have developed a general method of long DNA metabarcoding for analysis of omnivorous diets (Figure 1), through a diet study of the omnivorous European pond turtle (*Emys orbicularis*, L. 1758).

### 2.2 Study species

The European pond turtle is found in wetlands of Europe and North Africa and has been classified as “near threatened” (NT) according to the UICN Red List. In Switzerland, the species is listed as “critically endangered” (CR) on the Swiss Red List (Monney & Meyer, 2005). Previous studies investigating the feeding behaviour of the species using direct observations and microscopic examination have suggested that the animals have an omnivorous diet (Çicek & Ayaz, 2011; Ottonello et al., 2005, 2016, 2018). However, these techniques have several limitations, including the loss of information due to difficulties identifying prey and plant matter present in faeces. To our knowledge, no metabarcoding study exploring the European pond turtle diet have been conducted, and metabarcoding studies of reptile diets are scarce (Brown et al., 2012; Kartzinel & Pringle, 2015; Koizumi et al., 2017).

### 2.3 Collection of faecal samples and DNA extraction

European pond turtles were captured in April 2017 using conical fishing basket traps placed perpendicular to the banks (Cadi, 2003) in the natural reserve of Moulin de Vert (MDV; 46°10′46″N, 6°1′42″E, Canton of Geneva, Switzerland). Each trap was observed daily for a week and captured turtles were placed in individual containers without water for the night in order to collect faecal samples. Individuals were sexed, weighed, measured and released at the exact location at which they were captured. To prevent contamination of the samples, each container was cleaned with a 10% bleach solution, followed by 70% denatured ethanol. The captures were conducted after all necessary legal authorizations were acquired (see Acknowledgements). A total of 32 faecal samples were collected in the field. Each sample was stored in a plastic tube, and then placed in the freezer at −80°C until extractions were performed. In the laboratory, the faecal samples were homogenized and ground using liquid nitrogen. The risk of contamination between samples was minimized by decontaminating the mortar and pestle used for grinding in a bath of 10% bleach for 30 min, followed by rinsing with 70% denatured ethanol and UV irradiation for 15 min. Genomic DNA was then extracted from about 100 mg of the resulting powder with the Qiagen QIAamp PowerFecal DNA kit (Qiagen) according to the manufacturer's protocol. Other attempts with adapted CTAB DNA extraction protocols or another commercial kit (i.e., ISOLATE Fecal DNA kit from Bioline) did not achieve the performance of the Qiagen kit in terms of amplification quality of the extracted DNAs (data not shown). DNA quality and concentration were finally assessed with both a NanoDrop 1,000 Spectrophotometer and a Qubit 3.0 Fluorometer (both Thermo Fischer Scientific).

### 2.4 Feeding trial

Additionally, six captive European pond turtles were placed in individual containers in the laboratory, and food was withheld for 10 days to empty the digestive systems of the turtles (Devaux et al., 1996). Then, turtles were given diets consisting of a predetermined set of fishes and invertebrates (Table 1). The night after feeding, turtles were placed in dry containers (similar to those used in the field experiment), and multiple faecal samples were collected from each individual and subsequently homogenized. This procedure yielded six faecal samples with known diets, which were separately analysed as defined above.

**TABLE 1:** Dietary regimes (in g per ingested species) of six captive European pond turtles (*Emys orbicularis*, L. 1758) and their respective compositions determined using long metabarcoding analysis of faecal samples; the reference alignment length (bp) and percentage identity obtained after de novo assembly are provided

Ingested species	Emys_0			Emys_1			Emys_2			Emys_3			Emys_4			Emys_5		
	Amount consumed (g)	Percentage identity match	Reference alignment length (bp)	Amount consumed (g)	Percentage identity match	Reference alignment length (bp)	Amount consumed (g)	Percentage identity match	Reference alignment length (bp)	Amount consumed (g)	Percentage identity match	Reference alignment length (bp)	Amount consumed (g)	Percentage identity match	Reference alignment length (bp)	Amount consumed (g)	Percentage identity match	Reference alignment length (bp)
*Esox lucius*	20	98.52	323	—	—	—	19	97.83	366	10	99.78	566	—	—	—	13	100	499
*Oncorhynchus mykiss*	—	—	—	—	—	—	5	99.81	566	—	—	—	—	—	—	—	—	—
*Mus musculus*	—	—	—	3	98.52	244	6	99.77	567	15	99.13	350	—	—	—	5	99.3	296
*Chironomus salinarius*	5	97.84	573	8	98.86	636	5	98.71	588	5	99.6	248	8	98.54	397	—	—	—
*Gammarus pulex*	—	—	—	—	—	—	3	99.32	264	—	—	—	—	—	—	3	100	275

### 2.5 Mock community

A reference DNA database (mock community; MC) was set up using DNAs extracted from known components of the putative diet of the European pond turtle (Appendix S1: S1), which is composed of plants, macro‐invertebrates and fishes, according to the literature (Çicek & Ayaz, 2011; Ottonello et al., 2005, 2016, 2018). After individually grinding selected components of the MC in liquid nitrogen, genomic DNA was extracted from samples as described above. In order to highlight the limitations of both PCR amplification and bioinformatic analysis, two different types of mock community were prepared with the same DNA samples. (a) The first mock community (MC1) was a mixture of each DNA sample at a concentration of 10 ng/μl. PCRs were run for each primer set in triplicates with the mock community DNA mixture. (b) For the second mock community (MC2), each DNA sample was first individually amplified in triplicate with each primer set before pooling. For MC1 and MC2, DNA concentrations were determined as described above after purification of their respective pooled PCR products. Then, DNA amplicons from each component of both MCs were submitted to Sanger sequencing at Microsynth to validate species identity.

### 2.6 Primer selection and PCR amplification

Previously published primers used for the amplification of the large subunit of the ribulose‐1,5‐bisphosphate carboxylase gene (*rbcL*), the maturase K gene (*matK*), the 28S rRNA gene, the *trnL‐trnF* gene region in plants and a portion of the mitochondrial‐encoded cytochrome oxidase subunit I (*COI* or *COX1*) gene in animals were evaluated against DNA samples isolated from the mock community (Table 2). Several other primers pairs were tested (data not shown) but not retained, either because they were unable to amplify or lacked amplification specificity. Moreover, as live microorganisms such as bacteria and fungi are present in high numbers in the digestive systems of hosts, primer sets were also evaluated against DNAs of three bacterial (*Bacillus megaterium*, *Pseudomonas koreensis*, *Erwinia* sp.) and three fungal strains (*Aureobasidium pullulans*, *Trichoderma harzianum*, *Penicillium glabrum*), from our laboratory DNA collection. Primer pairs which amplified bacterial or fungal genes were then discarded. Primer pairs tested but not used were psbA3f (Sang et al., 1997) and trnHf‐05 (Tate & Simpson, 2003), matK 390‐F and matK 1326‐R (Cuénod et al., 2002), JK11 and JK14 (Aceto et al., 1999), COIF2 and COIR2 (Martinsen et al., 2008), C1‐J‐2182 (Simon et al., 1994) and TL2‐N‐3020 (Dobler & Müller, 2000), BF2 and BR2, BF2 and BR1, BF1 and BR2 (Elbrecht & Leese, 2017), ModRepCOI‐F and COI‐R, VertCOI_7194‐F and ModRepCOI‐R (Reeves et al., 2018), and Chmf4/Chmr4 (Che et al., 2012). This careful screening finally yielded seven primer pairs targeting three different types of plant genes and four primer pairs specific to *COI* sequences in vertebrates and invertebrates (Table 2). All PCRs were carried out using a 25‐μl reaction volume consisting of 5 µl MyTaq reaction buffer (Bioline), 2.5 µl of selected primers (0.5 μm final concentration), 2 U of MyTaq DNA polymerase (Bioline), 1 µl of DNA (concentrated at 10 ng/μl), and ultrapure sterile water up to completion volume. All PCRs were run in triplicate using the following reaction steps: an initial denaturation step at 95°C for 3 min, followed by 37 cycles of 95°C for 20 s, 52°C for 20 s and 72°C for 20 s, terminated by a final extension step of 20 s at 72°C. The annealing temperature was set to 52°C for all primer pairs except for mICOIintF and jgHCO2198 (54°C), and the primer pair Tab c and Tab f (56°C). These PCR programme conditions were established following the manufacturer's specifications and after optimization with DNAs from MCs, in order to yield the widest coverage at the family/species level while maintaining a high level of specificity at the amplification level. All PCR mixtures were prepared under a Biosan DNA/RNA UV‐Cleaner cabinet to avoid any contamination. Positive and negative controls were included.

**TABLE 2:** Primers used for amplification of the large subunit of ribulose‐1,5‐bisphophate carboxylase, maturase K, 28S rRNA, *trnL‐trnF* regions (for plants) and mitochondrial‐encoded cytochrome oxidase subunit I (for animals) gene

Prey taxon	DNA type	DNA region	Primer name	Forward/reverse primer	Primer sequence 5′–3′	References	Average length of amplified fragment (bp) in this study
Invertebrates	Mitochondrial	*COI*	mICOIintF	Forward	GGWACWGGWTGAACWGTWTAYCCYCC	Leray et al. (2013)	350
			jgHCO2198	Reverse	TAIACYTCIGGRTGICCRAARAAYCA	Leray et al. (2013)	
Invertebrates	Mitochondrial	*COI*	ODO_LCO1490d	Forward	TTTCTACWAACCAYAAAGATATTGG	Dijkstra et al. (2014)	650
			ODO_HCO2198d	Reverse	TAAACTTCWGGRTGTCCAAARAATCA	Dijkstra et al. (2014)	
Vertebrates	Mitochondrial	*COI*	COI‐CO2	Forward	AYTCAACAAATCATAAAGATATTGG	Che et al. (2012)	600
			COI‐CO4	Reverse	ACYTCRGGRTGACCAAAAAATCA	Che et al. (2012)	
Vertebrates	Mitochondrial	*COI*	Mod_RepCOI_F	Forward	TNTTYTCMACYAACCACAAAGA	Reeves et al. (2018)	650
			Mod_RepCOI_R	Reverse	TTCDGGRTGNCCRAARAATCA	Reeves et al. (2018)	
Plants	Plastid	*matK*	MatK−1RKIM‐f	Forward	ACCCAGTCCATCTGGAAATCTTGGTTC	K.‐J. Kim, pers. comm.	850
			MatK−3FKIM‐r	Reverse	CGTACAGTACTTTTGTGTTTACGAG	K.‐J. Kim, pers. comm.	
Plants	Plastid	*matK*	MatK−472‐f	Forward	CCCRTYCATCTGGAAATCTTGGTTC	Yu et al. (2011)	730
			MatK−1248‐r	Reverse	GCTRTRATAATGAGAAAGATTTCTGC	Yu et al. (2011)	
Plants	Plastid	*matK*	MatK−5r	Forward	GTTCTAGCACAAGAAAGTCG	Ford et al. (2009)	825
			MatK‐xf	Reverse	TAATTTACGATCAATTCATTC	Ford et al. (2009)	
Plants	Chloroplast	*rbcL*	rbcL a‐F	Forward	ATGTCACCACAAACAGAGACTAAAGC	Levin et al. (2003)	520
			rbcL a‐R	Reverse	GTAAAATCAAGTCCACCRCG	Kress and Erickson (2007)	
Plants	Chloroplast	*rbcL*	rbcL−1F	Forward	ATGTCACCACAAACAGAAAC	Fay et al. (1997)	680
			rbcL−724R	Reverse	TCGCATGTACCTGCAGTAGC	Olmstead et al. (1992)	
Plants	Chloroplast	28S rRNA	28KJ	Forward	GGCGGTAAATTCCGTCC	Cullings (1992)	630
			28C	Reverse	GCTATCCTGAGGGAAACTTC	Hamby and Zimmer (1988)	
Plants	Chloroplast	*trnL‐trnF*	Tab c	Forward	CGAAATCGGTAGACGCTACG	Tab erlet et al. (1991)	920
			Tab f	Reverse	ATTTGAACTGGTGACACGAG	Tab erlet et al. (1991)	

Furthermore, we designed host‐specific blocking primers, but the use of the blocking primer was not compatible with our metabarcoding approach (see explanations in Appendix S1: S2).

### 2.7 First upstream method validation

After metabarcode amplification, sequencing and bioinformatics were first validated upstream using in duplicate a synthetic mock community (sMC). In total, four distinct amplified genes from 14 organisms, which included plants, insects, fungi and bacteria (Table 3), were amplified using PCR, controlled using gel electrophoresis and their respective PCR products were pooled and purified with a Wizard Genomic Purification Kit (Promega). After measuring the DNA concentration with the Qubit 3.0 Fluorometer, the two pooled amplicons were diluted to 2 ng/μl and then fragmented using a protocol developed to create fragments with a mean size of 290 bp. This protocol utilizes the Covaris S2 focused‐ultrasonicator and can be applied to shear amplicons of a variety of lengths, ranging from 350 to 1,400 bp (Figure S3). The quality of sheared products was subsequently evaluated using a Tapestation 2,200 (Agilent).

**TABLE 3:** Species of invertebrates, plants, bacteria and fungi used as the synthetic mock community (sMC) for upstream method validation, with the primers used for amplification of ITS/5.8S rRNA, 16S, the *trnL‐trnF* region and the mitochondrial‐encoded cytochrome oxidase subunit I gene

Phylum	Species	Amplified gene	Primer name	Primer sequence 5′–3′	Reference	Median amplicon length (bp)	Post‐assembly median recovered contig length (bp)
Ascomycota	*Aureobasidium pullulans*	ITS1, 5.8S, ITS2	ITS4	TCCTCCGCTTATTGATATGC	White et al. (1990)	550	649
			ITS5	GGAAGTAAAAGTCGTAACAAGG	White et al. (1990)		
Ascomycota	*Trichoderma harzianum*	ITS1, 5.8S, ITS2	ITS4/5		White et al. (1990)	550	669
Ascomycota	*Penicillium glabrum*	ITS1, 5.8S, ITS2	ITS4/5		White et al. (1990)	550	645
Firmicutes	*Bacillus megaterium*	16S	27F/1492R	AGAGTTTGATCCTGGCTCAG	Hudson et al. (1998)	1,400	1,410
			1,492 R	CTACGGCTACCTTGTTACGA	Hudson et al. (1998)		
Proteobacteria	*Pseudomonas koreensis*	16S	27F/1492R		Hudson et al. (1998)	1,400	1,396
Proteobacteria	*Erwinia* sp.	16S	27F/1492R		Hudson et al. (1998)	1,400	1,400
Streptophyta	*Platanus occidentalis*	*trnL‐trnF*	Tabc/f	CGAAATCGGTAGACGCTACG	Tab erlet et al. (1991)	1,200	1,245
				ATTTGAACTGGTGACACGAG	Tab erlet et al. (1991)		
Streptophyta	*Chusquea culeou*	*trnL‐trnF*	Tabc/f		Tab erlet et al. (1991)	1,200	1,196
Arthropoda	*Aeshna juncea*	ITS1, 5.8S, ITS2	ITS4/5		Spadaro et al. (2012)	750	834
Arthropoda	*Aeshna cyanea*	ITS1, 5.8S, ITS2	ITS4/5		Spadaro et al. (2012)	750	837
Arthropoda	*Aeshna caerulea*	ITS1, 5.8S, ITS2	ITS4/5		Spadaro et al. (2012)	750	842
Arthropoda	*Aeshna juncea*	*COI*	ODO_LCO1490d	TTTCTACWAACCAYAAAGATATTGG	Dijkstra et al. (2014)	650	720
			ODO_HCO2198d	TAAACTTCWGGRTGTCCAAARAATCA	Dijkstra et al. (2014)		
Arthropoda	*Aeshna cyanea*	*COI*	ODO_LCO1490d/ ODO_HCO2198d		Dijkstra et al. (2014)	650	674
Arthropoda	*Aeshna caerulea*	*COI*	ODO_LCO1490d/ ODO_HCO2198d		Dijkstra et al. (2014)	650	717

### 2.8 Library and Illumina sequencing

Separate PCR amplifications of each DNA sample for all 11 barcodes (conducted in triplicate) were performed, and 9 µl of each amplification product was pooled together to create one sequencing library per sample and at the same time allow the analysis of a large number of samples in the most affordable and practical way. The resulting pool corresponding to each sample was then purified with the Wizard SV Gel and PCR Clean‐Up System and DNA concentration was assessed with a Qubit 3.0 Fluorometer. Amplicon pools were then diluted to a final concentration of 2 ng/μl and were finally fragmented to produce an average fragment size of 290 bp in AFA microtubes (Covaris) using an S2 focused‐ultrasonicator (Covaris), and following our established protocol given in Figure S3. Sequencing libraries were created using a TruSeq Nano DNA HT Library Prep Kit (Illumina) following the manufacturer's protocol. All samples were sequenced using an Illumina MiniSeq High Output run at 2 × 150 bp paired‐end read length, which reached a median sequencing depth of 106 Mb per sample.

### 2.9 Bioinformatic analysis

Illumina conversion software bcl2fastq2 version 2.20 was automatically run through the MiniSeq local run manager set with default parameters, in order to trim Illumina adapters and to demultiplex samples based on their respective index. The sequencing quality of the MiniSeq run was high, with 93% of the sequencing reads above the quality Phred score of 30. Nevertheless, cleaned reads were further evaluated for quality and adaptor contamination using fastqc (Andrews, 2010). An additional quality trimming of raw Illumina reads with trimmomatic 0.32 (Bogler et al., 2014) was evaluated on 10 samples, with stringent settings for base quality filtering, and was found not to be conclusive based on post de novo assembly results. Moreover, metaspades, the used de novo assembly software, comes with an “error correction read” process prior to contig assembly—i.e., “BayesHammer error correction tool,” which uses Bayesian subclustering to correct sequencing reads (Nikolenko et al., 2013). Following trimming and demultiplexing, cleaned sequencing reads were downloaded from the Illumina Basespace account. De novo assembly of sequencing data was separately carried out for each sample using the genome assembly software spades 3.11 (Nurk et al., 2017), with the metagenome assembly option (“metaSPAdes”) which includes the “error correction read” process prior to contig assembly. The parameters used in the software are described in Figure S4. Contigs smaller than 150 bp were removed, which represented between 0.17% and 10.34% of all contigs across all samples with an average of 2.86%. The resulting contigs files were analysed with the Basic Local Alignment Search Tool (blast) using blast+ (Camacho et al., 2009), searching the complete NCBI nucleotide (nt) database (command lines using blast + are described in Figure S3). Only the sequences of eukaryotes were conserved. Following the blast search characterized by a strong e‐value cut off (E‐value 1e−20) (Truelove et al., 2019), the five most significant matches (max_target_seq5) to the reference database for each of the query sequences were recorded. If only a single taxon was present in the top five and above 97.6% identity (see below for the level applied), the query was assigned directly to this taxon. If more than one reference taxon was present in the top five and above 97.6% identity, the query was assigned to the lowest taxonomic level that was shared by all taxa. In these specific cases (i.e., multiple taxa shared for a query sequence), the species identity was if possible confirmed without any ambiguity, thanks to the knowledge of biologists or botanists specialized in these studied sites. Finally, query sequences for which the best blast hit had less than 97.6% identity to any sequence were simply not considered. This threshold was determined following analysis of the sequences from both the mock communities and the captive feeding trials. The complete taxonomy of each species identity assignment per contig was completed using its respective TaxID and the ete3 toolkit software (Huerta‐Cepas et al., 2016). Finally, read abundance per contigs was determined using bowtie2 (Langmead & Salzberg, 2012) and samtools (Li et al., 2009) sequencing read alignment tools, plus an additional Perl script from multi‐metagenome (Albertsen et al., 2012) (command lines are described in Figure S4).

## 3 RESULTS

### 3.1 Upstream method validation

After sequencing, 14 amplicons (550–1400 bp in length) of the two MCs were entirely de novo assembled and all micro‐organism identities were successfully retrieved using NCBI blast. This confirmed that the proposed method could be performed on several different samples with DNA sequences (barcodes/amplicons) of different sizes, up to 1,400 bp and more.

### 3.2 Mock community

Two different mock communities with different amplification procedures (MC1 and MC2) were used to determine whether the differences in sequencing data were observed when DNAs were pooled before amplification (MC1) versus. amplified individually (MC2) (Table 4). All MC members within MC1 were identified to the species level, except for *Nymphaea alba*, which was assigned at the genus level only (*Nymphea* sp.). The average contig length for the pooled sample was 628 bp. For MC2, all MC members were also assigned to the species level and the average contig length was 425 bp. This experiment validated that the threshold was met for the determination of prey DNA from faeces to the species level. The sequence similarity requirement for species determination was >97.6% identity; below this threshold, analysis of the two MCs revealed false positives. This confirmed that if DNAs are pooled together (as in both MC1 and faecal samples), the established PCR configuration allows for the amplification of all the species present within the DNA mixture.

**TABLE 4:** Species composition of the two mock communities (MC1 and MC2)

MC_ID	Species	Percentage identity match	Reference alignment length (bp)
MC1	*Aeshna cyanea*	99.12	340
MC1	*Baetis rhodani*	99.34	603
MC1	*Bufotes viridis*	99.85	709
MC1	*Caenis* sp.	99.72	476
MC1	*Chironomus salinarius*	98.63	709
MC1	*Cloeon dipterum*	98.42	657
MC1	*Esox lucius*	99.43	572
MC1	*Gammarus pulex*	100.00	709
MC1	*Iris pseudacorus*	100.00	1,065
MC1	*Lycopus europaeus*	100.00	981
MC1	***Mentha spicata***	**97.70**	**957**
MC1	*Mus musculus*	99.84	682
MC1	*Notonecta glauca*	100.00	259
MC1	*Nuphar lutea*	100.00	642
MC1	***Nymphaea* sp.**	**99.38**	**161**
MC1	*Potamogeton perfoliatus*	100.00	495
MC1	*Radix balthica*	98.31	233
MC1	*Tinca tinca*	99.85	710
MC1	*Utricularia australis*	99.23	967
MC2	*Aeshna cyanea*	98.74	440
MC2	*Baetis rhodani*	99.18	540
MC2	*Bufotes viridis*	99.84	709
MC2	*Caenis* sp.	100	285
MC2	*Chironomus salinarius*	98.69	306
MC2	*Cloeon dipterum*	100	667
MC2	*Esox lucius*	99.28	279
MC2	*Gammarus pulex*	100	520
MC2	*Iris pseudacorus*	100	319
MC2	*Lycopus europaeus*	100	669
MC2	***Mentha spicata***	—	—
MC2	*Mus musculus*	99.31	436
MC2	*Notonecta glauca*	100	413
MC2	*Nuphar lutea*	100	342
MC2	*Nymphaea alba*	100	210
MC2	*Potamogeton perfoliatus*	100	226
MC2	*Radix balthica*	97.6	242
MC2	*Tinca tinca*	99.8	519
MC2	*Utricularia australis*	100	524

Note

Contig sequences (recovered amplicons) were obtained by de novo assembly with metaspades (Nurk et al., 2017) and queried using the Basic Local Alignment Search Tool (blast) and blast+ (Camacho et al., 2009), against the complete NCBI nucleotide (nt) database.

### 3.3 Captive feeding trial

Results of the analysis of faecal samples obtained by captive feeding trials demonstrated that every prey given to the European pond turtle was amplified and correctly assigned down to the species level. This produced a minimum identity threshold of 97.8% for the exact determination of prey species from DNA extracted from faeces and allowed for the allocation of identity to the species level. The average length of the reference alignment was 422 bp (Table 1).

### 3.4 Blocking primers

Without the use of any host‐specific blocking primers (that would have prevented host *COI* gene amplification), *Emys orbicularis* was formerly identified in only 12.5% of the samples, namely four out of 32. Across these four samples, we found that between 0.74% and 3.35% of raw sequencing paired‐end reads per sample aligned to the *Emys COI* gene contig, with an average of 1.78%. Considering the 32 samples totally, the average drops to 0.22% (Appendix S1: S5).

### 3.5 Qualitative analysis: Diet

**FIGURE 2:**
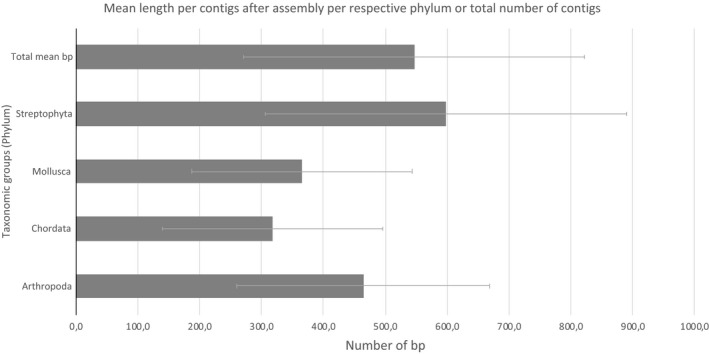
Mean length (bp; with *SD*) of the de novo assembly amplicons (contigs) assembled following long metabarcoding analysis of the 32 faecal samples of European pond turtles (*Emys orbicularis*). Total mean was calculated from a total of 233 contigs (each contig per sample is attributed to a unique OTU), whereas the other means were calculated per phylum

Metabarcoding analyses showed that all samples contained plant DNA, 46.9% of the samples contained vertebrate DNA and 84.4% of the samples contained macro‐invertebrate DNA. Most of the OTUs identified were assigned to a particular prey species (192 out of 233 OTUs; 82.4%); one invertebrate was determined to the order level only, another invertebrate to the family level and finally six plants to the genus level. In some cases, ecological information for Switzerland was available, and we were able to manually assign contig sequences to species known to be present in the area. For example, *Cladium* sp. was assigned to the species *Cladium mariscus* (Pohl, 1809) because it is known to be the only *Cladium* species living in Switzerland. Regarding *Betula* sp., the single *Betula* species found in this area and assigned in other samples was *Betula pubescens* (Ehrh, 1791), so we assigned this sequence to this species. For other genera (*Salix* sp., *Quercus* sp., *Picea* sp., *Carex* sp.), and for the family (Cecidomyiidae sp.) and order identified (Hemiptera sp.), it was not possible to identify the organism at a further precise level. Overall, this new long metabarcoding approach allowed us to allocate 82.4% of the amplified organisms to a precise species level. Thus, 34 different Arthropoda species were identified (with average amplicon length of 462 ± 204 bp), as well as three Mollusca species (365 ± 178 bp), three species of Chordata (318 ± 178 bp) and 28 plant species (598 ± 291 bp). A total of 68 different species were characterized within these results (mean 547 ± 274 bp) (Figure 2; Appendix S1: S6). No contamination was detected in the positive and negative controls.

### 3.6 Quantitative analysis: Read abundance

Finally, we determined the read abundance of all prey and plant species identified per sample, including the two MCs and feeding trial samples, in order to conclude whether we could use this information as a quantification indicator of metabarcoding diet (Appendix S1: S6). For instance, regarding plant species identified in our study, *Phragmites australis* was identified within the same sample MDV02 with three different barcode markers, namely *trnL‐trnF*, *matK* and *rbcL*, with 4.18%, 1.14% and 10.25% respectively of the total mapping reads. Another example, in the EMYS2 feeding control sample, we fed the individual with 19 g of *Esox lucius* and an average of 5 g of *Oncorhynchus mykiss*, *Mus musculus*, *Chironomus salinarius* and *Gammarus pulex*. Read abundance of *Esox lucius* was not significantly higher than the read abundance of the other prey taxa and was surprisingly even lower (Appendix S1: S5).

## 4 DISCUSSION

It has typically been assumed that prey DNA fragments in faecal samples were short and degraded as a result of the digestion by the host. This causes a major difficulty for the taxonomic identification of prey taxa using methods based on the analysis of DNA sequences within faecal samples (Deagle et al., 2006). For these reasons, previous protocols have used barcodes that target very short amplicons (<100 bp) (De Barba et al., 2014). However, the rate of DNA degradation in faeces may vary according to the identity of the ingested species. Indeed, in the present study, we found that plant parts (including seeds), bones and insect parts (such as legs and elytra) are only partially digested in the faecal samples. These observations suggest that prey DNA may not always be highly degraded and therefore enable the amplification of longer amplicons, thereby facilitating their identification down to the species level. Furthermore, the feeding trial experiment also demonstrated that the proposed long metabarcoding method can detect the DNA sequences of vertebrates and macro‐invertebrates fed to captive European pond turtles. In this case, prey could not be identified using direct observation or microscopy, because they were entirely digested. The method has been shown to be appropriate for the analysis of the diet of wild European pond turtle, and probably other species as well. DNA in faecal samples was not overly degraded, produced reliable results and allowed for the recovery of long amplicon sequences after de novo assembly. However, to accurately elucidate the real diet of the European pond turtle, the feeding trial should have contained plants (this information was unknown before this study). Use of mock communities, combined with samples obtained through captive feeding trials, proved to be essential to produce positive controls and validation data for parametrizing (threshold set up) bioinformatics pipelines. Finally, the various tests performed on samples collected throughout feeding trials and MCs enabled us to set a relatively high detection threshold at almost 98% identity. Without using any host‐specific blocking primers, we demonstrated that on average only 0.22% of raw sequencing paired‐end reads per sample aligned to *Emys* contigs. This is relatively weak and questions the usefulness of blocking primers related to the host DNA in metabarcoding diet studies from faeces. Indeed, it seemed, at least in the specific case of *Emys orbicularis*, that cell loss following cell renewal throughout the intestinal lumen of the host generates only a little or no DNA of amplifiable quality, as the *COI* gene of the host DNA was only identified in four out of 32 samples.

Regarding the qualitative analysis of faecal samples, the recovered amplicon lengths following de novo assembly (through contigs) varied between 168 and 1,379 bp (average of 546 bp); taxonomic resolution to the species level was reached for most sequences (82.4%). In previous diet analyses, taxonomic identification to the species level did not reach such high levels. For example, species were assigned for 75% of the fragments analysed in a study of red‐headed wood pigeon diet (Ando et al., 2013), for ≥60% when examining the diet of a bear (De Barba et al., 2014), and dietary analyses of the black wheatear detected the presence of animal DNA in 94 samples out of 112 using 18S, thus yielding 91 taxa from 21 orders of which 10% were assigned to the genus or species level (da Silva et al., 2019). Obtaining longer amplicons provides a well‐known advantage for our long metabarcoding approach because it enhances the precision of taxonomic identification (Heeger et al., 2018; Jamy et al., 2020; Liu et al., 2020; Piper et al., 2019; Porter & Golding, 2011). Furthermore, the approach developed here not only allowed us to amplify both barcodes of short to medium size, in cases of degraded DNA, but also and especially of long size (>500–650 bp and more), which is believed to increase the taxonomic affiliation at the species level. Indeed, shorter DNA fragments (e.g., 100 bp or less) are more likely to be sequenced, while longer DNA fragments provide a better taxonomic identification and resolution (Liu et al., 2020). Analyses made on degraded DNA samples demonstrated that few very informative barcodes such as *COI*, of shorter size between 135 bp (Hajibabaei et al., 2006) and 250 bp (Meusnier et al., 2008), can reliably identify animal species, if they target an appropriate placement within the larger barcode region (Elbrecht et al., 2019). Thus, longer amplicons generally increase the level of taxonomic assignment, and especially elevate the veracity and power of the results (absence of false positives; Piper et al., 2019). This method of long metabarcoding by short‐read sequencing (Illumina Platform) and de novo assembly has other advantages compared to the long‐read Pacific Bioscience sequencing platform used by Heeger et al. (2018) and Jamy et al. (2020). The method is more affordable and provides a higher level of sequencing depth. Moreover, our approach combining the use of different specific and universal primer pairs targeting both the same and unique gene (or gene portion) and several different genes as well, coupled with different targeted amplicon sizes (both long and short amplicons in case of highly degraded DNA), allowed us to amplify a large spectrum of species richness (da Silva et al., 2019) and to confirm some of the identified plant OTUs with redundancy of identification using many genes (*matK*, *rbcL* and 28S genes). Usually, species‐specific primers are used to amplify DNAs within a particular diet from faecal samples or gut contents (Leal et al., 2014; Pumarino et al., 2011; Wallinger et al., 2012). However, this approach is only useful if prior information regarding the diet of the studied animal is available and if the range of the diet is not too large (Moorhouse‐Gann et al., 2018). Thus, the use of multiple specific and universal primers in this study is an optimal method to determine complex diets, such as the diets of omnivorous animals such as the European pond turtle.

Nevertheless, the set of multiple primers that we developed cannot be considered as the optimal one for any diet study. In future research, depending on the diet or eDNA sample studied, additional primer pairs could be needed, to reach a higher level of discrimination at the species level (especially for plants), and to target longer amplicons. We hope this new approach can be an inspiration, and further developed and improved in many other diversity studies. The important sequencing depth reached with this approach (Illumina short‐read sequencing) allowed us to target a large number of different amplicons.

This approach maximizes taxonomic coverage and ensures that all potential target DNAs of prey species are amplified and correctly identified at the highest possible taxonomic level. However, even if the taxonomic assignment reached the species level for the majority of the samples, some plant taxa, such as the genera *Salix*, *Quercus*, *Picea* and *Carex*, were not identified to the species level despite the number of amplified genes produced and the seemingly adequate length of DNA sequences. Indeed, even by successfully coupling the identification of the genus *Carex* with several different amplified genes (i.e., *rbcL*, *trnL‐trnF*, *matK* and 28S), and long amplicons (>800–1,200 bp), taxonomic assignment at the species level was not possible and the final identification remained at the genus level, “*Carex* sp.” This may be caused by the difficultly associated with discriminating between closely related species or, more probably, by the incomplete nature of the NCBI database. Indeed, many species have not yet been added to this database (Kennedy et al., 2020). To identify the aforementioned plants to a higher taxonomic level, we recommend the elaboration of a local DNA sequence database, containing the plant species representing the known botanical diversity of the studied areas.

Unfortunately, one of the limitations of metabarcoding approaches is related to the status of the prey; indeed, it is not possible to determine whether adults, juveniles and/or eggs were consumed or if individuals were dead or alive. Moreover, plant fragments present in our samples could also be derived from prey. Indeed, some prey species of the European pond turtle are known to consume plants as a typical part of their own diets. The identification of certain plants may also be due to pollen contamination of the turtle's food. However, DNA present in faecal samples is usually degraded (Deagle et al., 2006), meaning that food items eaten by both prey and, subsequently, the predator, were degraded twice, making it unlikely that DNA fragments from this source could be detected. Furthermore, plant matter and seeds were present in large quantities in faecal samples, which confirmed the importance of combining direct observation with metabarcoding to validate the sequencing results.

Regarding the quantitative analysis, the amount of sequencing reads is a debatable indicator in the quantification of metabarcoding diets (Deagle et al., 2019). Indeed, the correlation between composition of the sample and sequence reads varies from none to strong. It remains to be shown whether biomass may be linked to read abundance as previously shown in copepods (Clarke et al., 2017; Hirai et al., 2015), in nematode communities (Schenk et al., 2019) and in below‐ground plants (Matesanz et al., 2019), while others failed to assess this link such as in zooplankton assemblages (Harvey et al., 2017). Given that metabarcoding relies primarily on barcode amplification, and that pairs of primers have different affinities for the multitude of targeted gene species amplified within a sample, variable amplification level completely biases the putative quantification of the identified species. In our samples, large differences in abundance are found for the same OTU within the same sample depending on the amplified barcode/gene, *rbcL* vs *matK*, *matK* vs *trnL‐trnF*, etc. For instance, in the MDV02 sample, the plant species *Phragmites australis* was identified with three contigs, namely *trnL‐trnF*, *matK* and *rbcL* with respectively 4.18%, 1.14% and 10.25% of the total mapping reads. Quantification of any dietary abundance data was therefore impossible with the current methodology. The abundance results of the species in the two MC samples confirmed this observation; they display large disparities depending on whether the DNAs were assembled before amplification or amplified separately in an equimolar manner. Additionally, according to the feeding controls, the metabarcoding analysis also revealed that quantification was not possible, as the relative ingested biomass does not correlate with the abundance of respective reads of each ingested prey. For instance, in EMYS2, we fed the individual with 19 g of *Esox lucius* and an average of 5 g of *Oncorhynchus mykiss*, *Mus musculus*, *Chironomus salinarius* and *Gammarus pulex*. Read abundance was not higher for *Esox lucius* as expected, but even lower compared to the other prey. Finally, a certain number of false negatives due to the lack of amplification of all the present species by the multiple pairs of primers used (both universal and specific) would also modify the proportion of biomass ingested and the numbers of reads.

Similarly, the efficiency of the digestion could have an impact on the detected DNA. In the present study, that is a diet analysis based on the extraction of DNAs from faeces, and verified by visual analysis (see also Ottonello et al., 2018), it was established that the DNA of some prey are excreted with higher integrity than others (e.g., intact seeds, bones, elytra of some beetles). Consequently, a prey can represent 95% of the food intake but its DNA, after extraction, would only represent 1% of the total faecal DNA, compared to a less digestible vegetable food (e.g., intact seeds) representing 5% of the food intake, which would represent 99% of the faecal DNA sample after extraction.

Finally, an additional bias preventing any relative quantitative evaluation when different barcodes are used is the number of copies of a barcoded gene within a targeted organism genome, and whether it is of nuclear, mitochondrial or chloroplast origin. When studying an omnivorous diet by metabarcoding analysis on stool samples, and with our current knowledge, it would be inappropriate to estimate a putative quantification of the prey ingested. Only qualitative analysis is reliable in this particular case.

The large species richness identified using the long metabarcoding approach proposed here is congruent with other molecular studies, which yielded high resolutions and an even greater richness regarding prey consumption, compared to histological analyses of the same samples (Soininen et al., 2015; Ando et al., 2013). To our knowledge, this is the first time that the metaspades software (Nurk et al., 2017) has been used for a long metabarcoding analysis, especially within a dietary study. We showed that this metagenome assembler is able to retrieve amplicons with a high confidence level and consequently provides an accurate taxonomic assignment.

Finally, this new long metabarcoding method with a short‐read sequencing approach, combining the use multiple primers pairs (da Silva et al., 2019) and de novo assembly, could be used as a universal, standardized method for studying complex diets, as well as in other complex eDNA analyses. Its high level of precision allows for improvements in studies of biodiversity assessments and trophic interactions, which would enhance our understanding of community ecology and ecosystem functioning.

## AUTHOR CONTRIBUTIONS

The first (C.D.) and second (J.C.) author contributed equally to this paper. C.D., S.U. and J.‐F.R. designed research, C.D. performed research (sampling and laboratory work) and analysed data (bioinformatics analyses), J.C. set up the methodology (shearing protocol, first upstream method validation, primer selection and design, sequencing and bioinformatics analyses), and J.C. and F.L. supervised laboratory work. C.D. wrote the manuscript and all other coauthors revised it.

## Supporting information

Appendix S1

## Data Availability

All raw sequencing reads for 40 metabarcodes (32 faecal samples from wild European pond turtles, six faecal samples from the feeding trial and two MCs) were registered in the Sequence Read Archive (SRA) database of the National Center for Biotechnology Information (NCBI) under Bioproject accession PRJNA546135 and the SRA accessions SRR9317490 to SRR9317499, SRR9317517 to SRR9317526, SRR9317540 to SRR9555, SRR9317564, SRR9317567 to SRR9317574 and SRR9317645.
