# Supplementary material for: A powerful long metabarcoding method for the determination of complex diets from faecal analysis of the European pond turtle (Emys orbicularis, L. 1758)
Source: Mol Ecol Resour. 2020 Nov 4;21(2):433–47. doi: 10.1111/1755-0998.13277 (PMC7821331; doi:10.1111/1755-0998.13277)
Supplement: Supplementary file 1 — Appendix S1 [file MEN-21-433-s001.docx]

**Supplemental Information for:**

**A powerful long metabarcoding method for determination of complex diets from faecal analysis of the European pond turtle (*Emys orbicularis*, L. 1758)**

Charlotte Ducotterd, Julien Crovadore, François Lefort, Jean-François Rubin, Sylvain Ursenbacher

**Table of Contents:**

| **Figure S1: Mock Community** | Page 2 |
| --- | --- |
| **Figure S2: Blocking primer** | Page 2 |
| **Figure S3: Covaris S2** | Page 3 |
| **Figure S4: Bioinformatic workflow** | Page 3 |
| **Figure S5: Number of reads** | Page 4 |
| **Figure S6: Faecal sample** | Page 13 |

**Figure S1.** Species comprising the putative diet of the European pond turtle (*Emys orbicularis*) were included in the Mock communities MC1 and MC2. The species have been selected according to the literature (Ottonello et al. 2005; Çiçek & Ayaz, 2011; Ottonello et al. 2016; Ottonello et al. 2018).

| **Phylum** | **Family** | **Species** | **English name** |
| --- | --- | --- | --- |
| Magnoliphyta | Iridaceae | *Iris pseudacorus* | Yellow iris |
| Magnoliphyta | Potamogetonaceae | *Potamogeton perfoliatus* | Gypswort |
| Magnoliphyta | Nymphaeaceae | *Nuphar lutea* | Yellow water-lily |
| Magnoliphyta | Nymphaeaceae | *Nymphaea alba* | European white water-lily |
| Magnoliphyta | Lamiaceae | *Lycopus europaeus* | Perfoliate pondweed |
| Magnoliphyta | Lentibulariaceae | *Utricularia australis* | Bladderwort |
| Magnoliphyta | Lamiaceae | *Mentha aquatica* | Aquatic mint |
| Arthropoda | Aeshnidae | *Aeshna cyanea* | Blue hawker |
| Arthorpoda | Baetidae | *Baetis rhodani* | Mayflies |
| Arthorpoda | Baetidae | *Cloeon dipterum* | Mayflies |
| Arthorpoda | Caenidae | *Caenis horaria* | Mayflies |
| Arthorpoda | Notonectoidea | *Notonecta glauca* | Backswimmer |
| Arthropoda | Chironomidae | *Chironomus salinarius* | Midges larvae |
| Arthorpoda | Gammaridae | *Gammarus pulex* | Amphipod crustacean |
| Mollusca | Lymnaeoidea | *Radix balthica* | Wandering snail |
| Chordata | Cyprinidae | *Tinca tinca* | Tench |
| Chordata | Esocidae | *Esox lucius* | Northern pike |
| Chordata | Bufonidae | *Bufotes viridis* | European green toad |
| Chordata | Muridae | *Mus musculus* | Grey mouse |

**Figure S2.** Host-specific blocking primer

Faecal samples contain degraded prey DNA but also contained high levels of high-quality host DNA. (Deagle et al., 2006). Predator DNA amplification may bias or prevent amplification of rare DNA sequences (Vestheim and Jarman, 2008; O’Rorke et al., 2012; Leray et al., 2013). Previous analyses (Roberson II et al., 2017) demonstrated that metabarcoding sequencing of faeces without a blocking primer, which prevents host DNA amplification, resulted in a high relative abundance of the host COI amplicons compare to target prey COI sequences.

Preliminary tests demonstrated that three of the four selected primers pairs targeting the COI region were perfectly amplifying *E. orbicularis* COI DNA. The seven selected primers pairs targeting plants were also tested against pond turtle DNA but did not produce amplicons as expected.

Consequently, based on an alignment of reference COI sequences of the European pond turtle, different blocking primers specific to *E. orbicularis* were developed, according to Vestheim and Jarman (2008). The aim was to block or limit amplification of COI sequences of the focal species. Turtle blocking primers overlapped with the 3’ end of the forward universal sequence, extending into *E. orbicularis*-specific sequences modified with a Spacer C3 at the 3’ end (Vestheim and Jarman, 2008). As two forward primers (ODO_LCO1490d and COI-CO2) were overlapping the same region of the COI sequence, a single blocking primer was needed for both. Sequences of the blocking primers were as follows: (1) COI-blkEmys1-3’c3 5’-ATAAAGATATTGGTACCCTCTATCT-C3-3’; (2) COI-blkEmys2-3’c3 5’-TGTATACCCCCCGCTAGCCGGAAAC-C3-3’. Turtle blocking primers were then rigorously evaluated against *E. orbicularis* DNA. COI-blkEmys1-3’c3 had proven to be effective by blocking 100% of the amplification of host DNA. On the other hand, COI-blkEmys2-3’c3 was totally ineffective despite numerous tests, even at a concentration a hundred times higher than its target primer pair in the reaction mixture. Finally, both were assessed against animal DNA from MC. Unfortunately, it turned out that under our established PCR conditions, COI-blkEmys1-3’c3 also partially inhibited the amplification of some DNA of target organisms. Therefore, the use of the blocking primer was not compatible with our metabarcoding approach.

**Figure S3.** Covaris S2 290 bp median size shearing protocol, applicable to a mixture of amplicons ranging from 350 to 1400 bp.

| **Amplicon length** | 350 to 1400 bp |
| --- | --- |
| **Median Target size** | 290 bp |
| **Duty Factor** | 10 |
| **Intensity** | 50 |
| **Peak/Display Power** | 23 |
| **Cycles/Burst** | 200 |
| **Mode** | Frequency sweeping |
| **T°C** | 5.5 |
| **Time (sec)** | 140 |

**Figure S4.** Bioinformatics workflow and command lines used with the software metaSPAdes v3.9.0 (Nurk et al., 2017; <http://cab.spbu.ru/software/spades>), BOWTIE2 (Langmead & Salzberg, 2012), SAMTOOLS (Li et al., 2009) and multi-metagenome (Albertsen et al., 2012) Perl script.

**Step 1**: Adapters removal and demultiplexing using automatic Illumina bcl2fastq2 conversion Software v2.20 through MiniSeq local run manager.

**Step 2**: Quality and adaptor contamination check using FastQC (Andrews, 2010).

**Step 3**: *De novo* assembly of the paired end reads using metaSPAdes.

./spades.py -o MetaSPAdesSampleX -meta -m 500 -t 72 -pe1-1 SampleX_R1.fastq.gz -pe1-2 SampleX _R2.fastq.gz

**Step 4**: Contigs mapping to NCBI server complete nucleotide database (nr/nt), using “BLAST+” (Camacho et al., 2009)

./blastn -db nt -query contigsSampleX.fasta -out resultscontigsSampleX.out -remote -outfmt ‘6 qseqid sseqid pident evalue staxids sscinames scomnames sskingdoms stitle std’ -max_target_seqs 5 -evalue 1e-20

**Step 5**: Identified species full taxonomy completion based on their respective TaxID and using ETE toolkit software (Huerta-Cepas et al., 2016).

./export PATH=~/anaconda_ete/bin:$PATH

./ete3 ncbiquery --search (*TaxIDs numbers list*) –info

**Step 6**: Determination of the reads abundance per contig/identified OTU

BOWTIE2 :

Index creation

bowtie2-build contigsSampleX.fasta contigsSampleX.btindex

Mapping

bowtie2 -x contigsSampleX.btindex -1 SampleX_R1.fastq -2 SampleX_R1.fastq -S SampleXbt2Map.sam

SAMTOOLS :

File conversion

samtools view -bS SampleXbt2Map.sam > SampleXbt2Map.bam

Alignment toward reference order

samtools sort SampleXbt2Map.bam -o SampleXbt2Map.sorted.bam

Obtaining "Depth" file

samtools depth SampleXbt2Map.sorted.bam > depthSampleX.txt

multi-metagenome

Convert file in .csv

perl [calc.coverage.in.bam.depth.pl](http://calc.coverage.in.bam.depth.pl) -i depthSampleX.txt -o coverage.lengthSampleX.csv

**Figure S5.** Analysis of faecal sample (N = 32) from the European pond turtle (*Emys orbicularis*), two mock communities and feeding trial samples. Sample ID, along with genus/species of each identified ingested preys and plants, targeted genes, contig length (bp), total reads number per sample, percentage of mapping reads and its respective number of reads.

| **Sample ID** | **Species** | **Targeted gene** | **Length (bp)** | **Total number of reads** | **% of mapping reads** | **Number of reads** |
| --- | --- | --- | --- | --- | --- | --- |
| EMYS0 | *Chironomus salinarius* | COI | 573 | 396575 | 0,45 | 1791 |
| EMYS0 | *Esox lucius* | COI | 323 | 396575 | 2,03 | 8042 |
| EMYS1 | *Chironomus salinarius* | COI | 636 | 338965 | 0,09 | 293 |
| EMYS1 | *Mus musculus* | COI | 244 | 338965 | 0,55 | 1875 |
| EMYS2 | *Esox lucius* | COI | 366 | 317877 | 0,03 | 93 |
| EMYS2 | *Chironomus salinarius* | COI | 588 | 317877 | 0,03 | 106 |
| EMYS2 | *Gammarus pulex* | COI | 221 | 317877 | 0,18 | 557 |
| EMYS2 | *Mus musculus* | COI | 567 | 317877 | 0,05 | 156 |
| EMYS2 | *Mus musculus* | COI | 218 | 317877 | 0,64 | 2045 |
| EMYS2 | *Oncorhynchus mykiss* | COI | 566 | 317877 | 0,53 | 1698 |
| EMYS3 | *Chironomus salinarius* | COI | 410 | 400717 | 0,27 | 1095 |
| EMYS3 | *Esox lucius* | COI | 566 | 400717 | 1,70 | 6801 |
| EMYS3 | *Mus musculus* | COI | 350 | 400717 | 0,02 | 96 |
| EMYS4 | *Chironomus salinarius* | COI | 397 | 327146 | 0,37 | 1218 |
| EMYS5 | *Esox lucius* | COI | 499 | 338415 | 0,57 | 1929 |
| EMYS5 | *Gammarus pulex* | COI | 275 | 338415 | 0,65 | 2188 |
| EMYS5 | *Mus musculus* | COI | 296 | 338415 | 0,01 | 49 |
| MC1 | *Iris pseudacorus* | trnL - trnF | 1065 | 355086 | 0,62 | 2208 |
| MC1 | *Iris pseudacorus* | matK | 989 | 355086 | 2,90 | 10307 |
| MC1 | *Iris pseudacorus* | rbcL | 261 | 355086 | 0,34 | 1219 |
| MC1 | *Utricularia australis* | trnL - trnF | 967 | 355086 | 0,08 | 298 |
| MC1 | *Mentha spicata* | matK | 957 | 355086 | 0,88 | 3135 |
| MC1 | *Mentha spicata* | rbcL | 957 | 355086 | 0,73 | 2583 |
| MC1 | *Tinca tinca* | COI | 710 | 355086 | 0,62 | 2187 |
| MC1 | *Chironomus salinarius* | COI | 709 | 355086 | 2,06 | 7309 |
| MC1 | *Gammarus pulex* | COI | 709 | 355086 | 0,27 | 945 |
| MC1 | *Bufotes viridis* | COI | 709 | 355086 | 0,07 | 246 |
| MC1 | *Mus musculus* | COI | 682 | 355086 | 0,09 | 327 |
| MC1 | *Cloeon dipterum* | COI | 657 | 355086 | 0,04 | 126 |
| MC1 | *Nuphar lutea* | rbcL | 270 | 355086 | 0,16 | 566 |
| MC1 | *Nuphar lutea* | matK | 636 | 355086 | 4,58 | 16265 |
| MC1 | *Baetis rhodani* | COI | 603 | 355086 | 0,01 | 24 |
| MC1 | *Esox lucius* | COI | 572 | 355086 | 0,03 | 108 |
| MC1 | *Potamogeton perfoliatus* | trnL - trnF | 1120 | 355086 | 0,50 | 1793 |
| MC1 | *Potamogeton perfoliatus* | rbcL | 635 | 355086 | 4,89 | 17368 |
| MC1 | *Potamogeton perfoliatus* | matK | 588 | 355086 | 0,74 | 2625 |
| MC1 | *Caenis horaria* | COI | 476 | 355086 | 0,01 | 37 |
| MC1 | *Aeshna cyanea* | COI | 340 | 355086 | 0,01 | 23 |
| MC1 | *Notonecta glauca* | COI | 259 | 355086 | 0,21 | 735 |
| MC1 | *Radix balthica* | COI | 233 | 355086 | 0,00 | 9 |
| MC2 | *Aeshna cyanea* | COI | 440 | 374942 | 0,27 | 997 |
| MC2 | *Baetis rhodani* | COI | 540 | 374942 | 0,02 | 77 |
| MC2 | *Bufotes viridis* | COI | 709 | 374942 | 0,56 | 2100 |
| MC2 | *Caenis horaria* | COI | 285 | 374942 | 0,04 | 151 |
| MC2 | *Chironomus salinarius* | COI | 306 | 374942 | 2,09 | 7847 |
| MC2 | *Cloeon dipterum* | COI | 667 | 374942 | 0,29 | 1086 |
| MC2 | *Esox lucius* | COI | 279 | 374942 | 0,21 | 804 |
| MC2 | *Gammarus pulex* | COI | 520 | 374942 | 1,61 | 6029 |
| MC2 | *Iris pseudacorus* | matK | 391 | 374942 | 7,86 | 29460 |
| MC2 | *Iris pseudacorus* | rbcL | 230 | 374942 | 1,46 | 5467 |
| MC2 | *Iris pseudacorus* | trnL - trnF | 229 | 374942 | 2,98 | 11169 |
| MC2 | *Lycopus europaeus* | trnL - trnF | 669 | 374942 | 4,93 | 18486 |
| MC2 | *Lycopus europaeus* | matK | 560 | 374942 | 4,02 | 15089 |
| MC2 | *Mus musculus* | COI | 436 | 374942 | 0,36 | 1352 |
| MC2 | *Notonecta glauca* | COI | 413 | 374942 | 0,05 | 181 |
| MC2 | *Nuphar lutea* | matK | 166 | 374942 | 8,22 | 30838 |
| MC2 | *Nymphaea alba* | rbcL | 166 | 374942 | 3,41 | 12781 |
| MC2 | *Potamogeton perfoliatus* | trnL - trnF | 226 | 374942 | 1,26 | 4726 |
| MC2 | *Potamogeton perfoliatus* | rbcL | 197 | 374942 | 2,05 | 7692 |
| MC2 | *Radix balthica* | COI | 242 | 374942 | 0,02 | 59 |
| MC2 | *Tinca tinca* | COI | 519 | 374942 | 0,93 | 3474 |
| MC2 | *Utricularia australis* | matK | 524 | 374942 | 4,76 | 17840 |
| MDV01 | Host - *Emys orbicularis* | COI | 712 | 282083 | 0,74 | 2085 |
| MDV05 | Host - *Emys orbicularis* | COI | 366 | 351453 | 3,35 | 11777 |
| MDV23 | Host - *Emys orbicularis* | COI | 709 | 253462 | 1,37 | 3468 |
| MDV35 | Host - *Emys orbicularis* | COI | 365 | 677104 | 1,68 | 11369 |
| MDV01 | *Bufo bufo* | COI | 217 | 564166 | 1,07 | 6009 |
| MDV01 | *Endochironomus tendens* | COI | 706 | 564166 | 0,01 | 65 |
| MDV01 | *Phaenopsectra punctipes* | COI | 233 | 564166 | 0,01 | 44 |
| MDV01 | *Phragmites australis* | trnL - trnF | 1041 | 564166 | 0,45 | 2566 |
| MDV01 | *Phragmites australis* | rbcL | 751 | 564166 | 0,99 | 5587 |
| MDV01 | *Streblotrichum convolutum* | rbcL | 650 | 564166 | 2,46 | 13866 |
| MDV01 | *Limnephilus flavicornis* | COI | 709 | 564166 | 0,06 | 335 |
| MDV02 | *Alnus glutinosa* | rbcL | 421 | 636104 | 4,11 | 26174 |
| MDV02 | *Alnus glutinosa* | trnL - trnF | 1055 | 636104 | 0,06 | 367 |
| MDV02 | *Betula pubescens* | matK | 834 | 636104 | 0,23 | 1492 |
| MDV02 | *Populus alba* | rbcL | 584 | 636104 | 6,45 | 41018 |
| MDV02 | *Populus nigra* | rbcL | 281 | 636104 | 7,45 | 47395 |
| MDV02 | *Juncus effusus* | 28S | 364 | 636104 | 2,15 | 13703 |
| MDV02 | *Juncus effusus* | 28S | 272 | 636104 | 3,28 | 20855 |
| **MDV02** | ***Phragmites australis*** | **trnL - trnF** | **1041** | **636104** | **4,18** | **26606** |
| **MDV02** | ***Phragmites australis*** | **matK** | **889** | **636104** | **1,14** | **7258** |
| **MDV02** | ***Phragmites australis*** | **rbcL** | **751** | **636104** | **10,25** | **65208** |
| MDV03 | *Alnus alnobetula* | 18S | 548 | 705948 | 7,96 | 56197 |
| MDV03 | *Ilyocoris cimicoides* | COI | 366 | 705948 | 0,09 | 635 |
| MDV03 | *Asellus aquaticus* | COI | 365 | 705948 | 0,35 | 2470 |
| MDV03 | *Populus alba* | rbcL | 307 | 705948 | 0,38 | 2655 |
| MDV03 | *Brachytron pratense* | COI | 385 | 705948 | 3,56 | 25151 |
| MDV03 | *Pyrrhosoma nymphula* | COI | 706 | 705948 | 0,01 | 102 |
| MDV03 | *Carex sp.* | 28S | 285 | 705948 | 6,63 | 46833 |
| MDV03 | *Carex sp.* | rbcL | 318 | 705948 | 5,20 | 36707 |
| MDV03 | *Juncus effusus* | 28S | 312 | 705948 | 7,38 | 52113 |
| MDV03 | *Phragmites australis* | trnL - trnF | 257 | 705948 | 0,00 | 10 |
| MDV03 | *Phragmites australis* | rbcl | 426 | 705948 | 6,92 | 48830 |
| MDV03 | *Phragmites australis* | matK | 891 | 705948 | 0,48 | 3356 |
| MDV03 | *Castor fiber* | COI | 259 | 705948 | 0,01 | 105 |
| MDV03 | *Limnephilus flavicornis* | COI | 709 | 705948 | 1,23 | 8675 |
| MDV04 | *Potamogeton perfoliatus* | trnL - trnF | 1056 | 717992 | 0,03 | 196 |
| MDV04 | *Bufo bufo* | COI | 328 | 717992 | 0,15 | 1081 |
| MDV04 | *Alnus alnobetula* | 28S | 472 | 717992 | 1,97 | 14166 |
| MDV04 | *Betula pubescens* | matK | 547 | 717992 | 0,01 | 67 |
| MDV04 | *Betula pubescens* | trnL - trnF | 889 | 717992 | 0,10 | 701 |
| MDV04 | *Quercus sp.* | trnL - trnF | 922 | 717992 | 0,05 | 371 |
| MDV04 | *Quercus sp.* | rbcL | 627 | 717992 | 3,56 | 25575 |
| MDV04 | *Quercus sp.* | matK | 889 | 717992 | 0,15 | 1056 |
| MDV04 | *Ilyocoris cimicoides* | COI | 727 | 717992 | 5,41 | 38826 |
| MDV04 | *Salix sp.* | 28S | 492 | 717992 | 4,04 | 29017 |
| MDV04 | *Salix sp.* | matK | 594 | 717992 | 8,42 | 60458 |
| MDV04 | *Phragmites australis* | matK | 922 | 717992 | 0,13 | 927 |
| MDV04 | *Phragmites australis* | rbcL | 738 | 717992 | 6,89 | 49443 |
| MDV04 | *Phragmites australis* | trnL - trnF | 1040 | 717992 | 1,51 | 10859 |
| MDV04 | *Limnephilus flavicornis* | COI | 707 | 717992 | 0,03 | 180 |
| MDV05 | *Caenis horaria* | COI | 233 | 702906 | 0,00 | 33 |
| MDV05 | *Betula pubescens* | rbcL | 587 | 702906 | 2,01 | 14150 |
| MDV05 | *Ilyocoris cimicoides* | COI | 365 | 702906 | 0,01 | 50 |
| MDV05 | *Anax imperator* | COI | 365 | 702906 | 0,01 | 45 |
| MDV05 | *Phragmites australis* | matK | 891 | 702906 | 0,71 | 4978 |
| MDV05 | *Phragmites australis* | rbcL | 751 | 702906 | 6,02 | 42323 |
| MDV05 | *Phragmites australis* | trnL - trnF | 1379 | 702906 | 1,60 | 11220 |
| MDV05 | *Limnephilus flavicornis* | COI | 709 | 702906 | 0,02 | 115 |
| MDV06 | *Alnus alnobetula* | 28S | 548 | 750912 | 9,19 | 68979 |
| MDV06 | *Betula pubescens* | trnL - trnF | 901 | 750912 | 0,04 | 335 |
| MDV06 | *Ilyocoris cimicoides* | COI | 366 | 750912 | 0,01 | 77 |
| MDV06 | *Carex sp.* | rbcL | 426 | 750912 | 2,58 | 19342 |
| MDV06 | *Phragmites australis* | rbcL | 580 | 750912 | 6,39 | 47949 |
| MDV06 | *Phragmites australis* | matK | 898 | 750912 | 0,62 | 4619 |
| MDV06 | *Phragmites australis* | trnL - trnF | 1041 | 750912 | 0,71 | 5318 |
| MDV06 | *Limnephilus vittatus* | COI | 316 | 750912 | 0,04 | 295 |
| MDV07 | *Alnus subcordata* | 28S | 176 | 765398 | 2,35 | 17990 |
| MDV07 | *Hypnum cupressiforme* | rbcL | 599 | 765398 | 0,62 | 4712 |
| MDV07 | *Populus alba* | matK | 370 | 765398 | 7,47 | 57165 |
| MDV07 | *Populus nigra* | rbcL | 455 | 765398 | 0,00 | 30 |
| MDV07 | *Carex sp.* | 28S | 541 | 765398 | 5,41 | 41402 |
| MDV07 | *Phragmites australis* | rbcl | 774 | 765398 | 12,29 | 94065 |
| MDV07 | *Phragmites australis* | trnL - trnF | 1041 | 765398 | 0,23 | 1750 |
| MDV08 | *Gammarus fossarum* | COI | 365 | 745676 | 0,03 | 191 |
| MDV08 | *Chironomus pallidivittatus* | COI | 423 | 745676 | 0,01 | 45 |
| MDV08 | *Endochironomus tendens* | COI | 706 | 745676 | 0,02 | 133 |
| MDV08 | *Alnus alnobetula* | 28S | 696 | 745676 | 7,15 | 53330 |
| MDV08 | *Asellus aquaticus* | COI | 387 | 745676 | 0,00 | 18 |
| MDV08 | *Carex sp.* | trnL - trnF | 451 | 745676 | 0,00 | 32 |
| MDV08 | *Carex sp.* | rbcL | 398 | 745676 | 1,19 | 8902 |
| MDV08 | *Phragmites australis* | matK | 890 | 745676 | 0,31 | 2307 |
| MDV08 | *Phragmites australis* | rbcL | 591 | 745676 | 5,34 | 39839 |
| MDV08 | *Phragmites australis* | trnL - trnF | 1040 | 745676 | 1,25 | 9357 |
| MDV08 | *Barbula unguiculata* | rbcL | 579 | 745676 | 1,05 | 7795 |
| MDV08 | *Limnephilus flavicornis* | COI | 706 | 745676 | 0,60 | 4486 |
| MDV09 | *Bufo bufo* | COI | 207 | 708078 | 0,00 | 10 |
| MDV09 | *Alnus alnobetula* | 28S | 579 | 708078 | 3,21 | 22749 |
| MDV09 | *Quercus sp.* | matK | 266 | 708078 | 0,00 | 16 |
| MDV09 | *Quercus sp.* | trnL - trnF | 915 | 708078 | 0,05 | 360 |
| MDV09 | *Populus alba* | rbcL | 284 | 708078 | 0,60 | 4225 |
| MDV09 | *Carex sp.* | rbcL | 543 | 708078 | 1,31 | 9261 |
| MDV09 | *Phragmites australis* | rbcL | 548 | 708078 | 6,08 | 43052 |
| MDV09 | *Phragmites australis* | matK | 891 | 708078 | 0,55 | 3917 |
| MDV09 | *Pleurochaete squarrosa* | trnL - trnF | 504 | 708078 | 0,54 | 3790 |
| MDV09 | *Pottiopsis caespitosa* | rbcL | 620 | 708078 | 3,00 | 21229 |
| MDV10 | *Cloeon dipterum* | COI | 706 | 752412 | 0,03 | 254 |
| MDV10 | *Alnus glutinosa* | rbcL | 265 | 752412 | 8,70 | 65449 |
| MDV10 | *Betula pubescens* | matK | 889 | 752412 | 0,27 | 2039 |
| MDV10 | *Radix auricularia* | COI | 263 | 752412 | 0,01 | 53 |
| MDV10 | *Pheosia tremula* | COI | 212 | 752412 | 0,00 | 22 |
| MDV10 | *Salix sp.* | rbcL | 282 | 752412 | 4,74 | 35654 |
| MDV11 | *Equisetum hyemale* | trnL - trnF | 275 | 791092 | 0,01 | 45 |
| MDV11 | *Alnus glutinosa* | rbcL | 416 | 791092 | 10,16 | 80345 |
| MDV11 | *Alnus glutinosa* | trnL - trnF | 961 | 791092 | 1,37 | 10825 |
| MDV11 | *Ilyocoris cimicoides* | COI | 709 | 791092 | 0,16 | 1227 |
| MDV11 | *Salix sp.* | trnL - trnF | 232 | 791092 | 0,00 | 18 |
| MDV11 | *Salix sp.* | rbcL | 338 | 791092 | 8,20 | 64850 |
| MDV11 | *Calopteryx virgo* | COI | 712 | 791092 | 0,01 | 52 |
| MDV11 | *Coenagrion pulchellum* | COI | 710 | 791092 | 0,77 | 6096 |
| MDV11 | *Carex sp.* | 28S | 591 | 791092 | 0,38 | 3033 |
| MDV11 | *Phragmites australis* | matK | 888 | 791092 | 0,03 | 209 |
| MDV11 | *Phragmites australis* | rbcL | 743 | 791092 | 6,40 | 50639 |
| MDV11 | *Phragmites australis* | trnL - trnF | 1041 | 791092 | 0,02 | 143 |
| MDV11 | *Tortella tortuosa* | rbcL | 502 | 791092 | 0,75 | 5944 |
| MDV11 | *Pleurochaete squarrosa* | trnL - trnF | 635 | 791092 | 0,02 | 132 |
| MDV11 | *Limnephilus flavicornis* | COI | 712 | 791092 | 10,21 | 80764 |
| MDV12 | *Cyprinus carpio* | COI | 300 | 653550 | 7,95 | 51952 |
| MDV12 | *Alnus glutinosa* | rbcL | 421 | 653550 | 1,25 | 8139 |
| MDV12 | *Betula pubescens* | trnL - trnF | 497 | 653550 | 0,03 | 168 |
| MDV12 | *Notonecta glauca* | COI | 266 | 653550 | 0,00 | 18 |
| MDV12 | *Bithynia tentaculata* | COI | 364 | 653550 | 0,00 | 26 |
| MDV12 | *Carex sp.* | trnL - trnF | 269 | 653550 | 0,00 | 23 |
| MDV12 | *Carex sp.* | rbcL | 676 | 653550 | 2,76 | 18010 |
| MDV12 | *Phragmites australis* | trnL - trnF | 1050 | 653550 | 0,66 | 4292 |
| MDV12 | *Phragmites australis* | matK | 890 | 653550 | 0,53 | 3451 |
| MDV12 | *Phragmites australis* | rbcL | 790 | 653550 | 8,40 | 54887 |
| MDV12 | *Pleurochaete squarrosa* | trnL - trnF | 502 | 653550 | 0,09 | 600 |
| MDV12 | *Tortella tortuosa* | trnL - trnF | 669 | 653550 | 2,07 | 13560 |
| MDV12 | *Limnephilus flavicornis* | COI | 436 | 653550 | 0,18 | 1174 |
| MDV14 | *Cricotopus bicinctus* | COI | 229 | 673182 | 0,01 | 45 |
| MDV14 | *Quercus sp.* | 28S | 543 | 673182 | 15,10 | 101677 |
| MDV14 | *Quercus sp.* | trnL - trnF | 376 | 673182 | 0,01 | 70 |
| MDV14 | *Sympetrum striolatum* | COI | 364 | 673182 | 0,01 | 72 |
| MDV14 | *Carex sp.* | 28S | 282 | 673182 | 5,50 | 37017 |
| MDV14 | *Carex sp.* | rbcL | 655 | 673182 | 14,79 | 99531 |
| MDV14 | *Cladium mariscus* | trnL - trnF | 870 | 673182 | 0,03 | 207 |
| MDV14 | *Juncus effusus* | 28S | 31 | 673182 | 3,02 | 20340 |
| MDV14 | *Phragmites australis* | trnL - trnF | 975 | 673182 | 0,23 | 1522 |
| MDV14 | *Phragmites australis* | rbcL | 426 | 673182 | 14,79 | 99534 |
| MDV15 | *Alnus alnobetula* | 28S | 474 | 758966 | 3,76 | 28512 |
| MDV15 | *Quercus sp.* | rbcL | 601 | 758966 | 4,54 | 34472 |
| MDV15 | *Quercus sp.* | trnL - trnF | 336 | 758966 | 0,00 | 36 |
| MDV15 | *Phragmites australis* | rbcL | 818 | 758966 | 7,89 | 59882 |
| MDV15 | *Phragmites australis* | trnL - trnF | 1041 | 758966 | 0,19 | 1466 |
| MDV15 | *Limnephilus flavicornis* | COI | 271 | 758966 | 0,00 | 34 |
| MDV16 | *Bufo bufo* | COI | 708 | 775452 | 0,02 | 144 |
| MDV16 | *Salix sp.* | rbcL | 347 | 775452 | 6,09 | 47254 |
| MDV16 | *Phragmites australis* | trnL - trnF | 1041 | 775452 | 0,06 | 443 |
| MDV16 | *Phragmites australis* | rbcL | 785 | 775452 | 5,27 | 40833 |
| MDV16 | *Limnephilus flavicornis* | COI | 709 | 775452 | 0,03 | 264 |
| MDV17 | *Cricotopus triannulatus* | COI | 709 | 821384 | 0,04 | 289 |
| MDV17 | *Equisetum ramosissimum* | rbcL | 234 | 821384 | 0,00 | 17 |
| MDV17 | *Betula pubescens* | trnL - trnF | 823 | 821384 | 0,19 | 1520 |
| MDV17 | *Quercus sp.* | rbcL | 922 | 821384 | 0,03 | 264 |
| MDV17 | *Asellus aquaticus* | COI | 365 | 821384 | 0,01 | 109 |
| MDV17 | *Salix sp.* | rbcL | 306 | 821384 | 1,00 | 8210 |
| MDV17 | *Carex sp.* | rbcL | 232 | 821384 | 0,24 | 1954 |
| MDV17 | *Phragmites australis* | trnL - trnF | 1040 | 821384 | 3,63 | 29848 |
| MDV17 | *Limnephilus flavicornis* | COI | 709 | 821384 | 0,27 | 2195 |
| MDV18 | *Potamogeton perfoliatus* | trnL - trnF | 984 | 730360 | 0,02 | 145 |
| MDV18 | *Agrilus angustulus* | COI | 179 | 730360 | 9,99 | 72990 |
| MDV18 | *Alnus incana* | 28S | 493 | 730360 | 10,81 | 78926 |
| MDV18 | *Ilyocoris cimicoides* | COI | 365 | 730360 | 9,28 | 67759 |
| MDV18 | *Bithynia tentaculata* | COI | 281 | 730360 | 0,01 | 46 |
| MDV18 | *Salix sp.* | 28S | 241 | 730360 | 0,00 | 20 |
| MDV18 | *Carex sp.* | rbcL | 255 | 730360 | 7,14 | 52140 |
| MDV18 | *Cladium sp.* | rbcL | 868 | 730360 | 0,69 | 5011 |
| MDV18 | *Phragmites australis* | rbcL | 429 | 730360 | 8,49 | 62036 |
| MDV18 | *Phragmites australis* | matK | 890 | 730360 | 0,09 | 662 |
| MDV18 | *Phragmites australis* | trnL - trnF | 975 | 730360 | 1,21 | 8822 |
| MDV18 | *Tortella tortuosa* | 28S | 560 | 730360 | 1,75 | 12772 |
| MDV19 | *Phragmites australis* | trnL - trnF | 1042 | 677986 | 2,06 | 13937 |
| MDV19 | *Phragmites australis* | matK | 889 | 677986 | 0,28 | 1910 |
| MDV19 | *Phragmites australis* | rbcL | 784 | 677986 | 10,73 | 72761 |
| MDV19 | *Limnephilus flavicornis* | COI | 709 | 677986 | 0,06 | 416 |
| MDV21 | *Alnus incana* | 28S | 579 | 766000 | 6,67 | 51104 |
| MDV21 | *Armadillidium nasatum* | COI | 224 | 766000 | 0,80 | 6145 |
| MDV21 | *Populus alba* | rbcL | 262 | 766000 | 3,02 | 23158 |
| MDV21 | *Phragmites australis* | rbcL | 385 | 766000 | 21,98 | 168331 |
| MDV21 | *Phragmites australis* | trnL - trnF | 252 | 766000 | 0,71 | 5427 |
| MDV22 | *Gammarus fossarum* | COI | 263 | 539366 | 0,01 | 29 |
| MDV22 | *Bufo bufo* | COI | 208 | 539366 | 0,01 | 28 |
| MDV22 | *Donacia clavipes* | COI | 213 | 539366 | 0,00 | 18 |
| MDV22 | *Betula sp.* | trnL - trnF | 634 | 539366 | 1,43 | 7726 |
| MDV22 | *Hypnum cupressiforme* | trnL - trnF | 249 | 539366 | 0,03 | 187 |
| MDV22 | *Populus alba* | rbcL | 744 | 539366 | 1,07 | 5752 |
| MDV22 | *Phragmites australis* | trnL - trnF | 1041 | 539366 | 1,66 | 8939 |
| MDV22 | *Phragmites australis* | matK | 889 | 539366 | 0,08 | 414 |
| MDV22 | *Phragmites australis* | rbcL | 745 | 539366 | 12,33 | 66501 |
| MDV23 | *Potamogeton perfoliatus* | matK | 793 | 506924 | 0,10 | 526 |
| MDV23 | *Caenis horaria* | COI | 288 | 506924 | 0,00 | 19 |
| MDV23 | *Alnus alnobetula* | 28S | 579 | 506924 | 9,54 | 48346 |
| MDV23 | *Betula pubescens* | rbcL | 308 | 506924 | 3,43 | 17380 |
| MDV23 | *Bithynia tentaculata* | COI | 709 | 506924 | 0,09 | 435 |
| MDV23 | *Coenagrion pulchellum* | COI | 715 | 506924 | 0,48 | 2433 |
| MDV23 | *Carex sp.* | rbcL | 398 | 506924 | 6,43 | 32591 |
| MDV23 | *Phragmites australis* | trnL - trnF | 547 | 506924 | 6,49 | 32891 |
| MDV23 | *Phragmites australis* | rbcL | 271 | 506924 | 0,00 | 10 |
| MDV23 | *Pleurochaete squarrosa* | trnL - trnF | 274 | 506924 | 0,00 | 15 |
| MDV23 | *Limnephilus flavicornis* | COI | 365 | 506924 | 0,07 | 376 |
| MDV24 | *Alnus alnobetula* | 28S | 729 | 667950 | 11,09 | 74053 |
| MDV24 | *Quercus sp.* | rbcL | 344 | 667950 | 1,76 | 11750 |
| MDV24 | *Bithynia tentaculata* | COI | 365 | 667950 | 0,01 | 40 |
| MDV24 | *Populus alba* | rbcL | 637 | 667950 | 7,74 | 51722 |
| MDV24 | *Carex sp.* | rbcL | 398 | 667950 | 10,93 | 72983 |
| MDV24 | *Phragmites australis* | rbcL | 639 | 667950 | 10,45 | 69825 |
| MDV25 | *Phragmites australis* | rbcL | 1040 | 492756 | 0,29 | 1445 |
| MDV25 | *Limnephilus flavicornis* | COI | 622 | 492756 | 0,00 | 10 |
| MDV26 | *Populus alba* | rbcL | 629 | 758900 | 11,24 | 85303 |
| MDV26 | *Carex sp.* | rbcL | 398 | 758900 | 8,55 | 64906 |
| MDV26 | *Carex sp.* | trnL - trnF | 241 | 758900 | 0,00 | 18 |
| MDV26 | *Phragmites australis* | trnL - trnF | 1041 | 758900 | 1,00 | 7581 |
| MDV26 | *Phragmites australis* | rbcL | 399 | 758900 | 11,62 | 88180 |
| MDV26 | *Pleurochaete squarrosa* | trnL - trnF | 503 | 758900 | 1,61 | 12189 |
| MDV26 | *Tortella tortuosa* | trnL - trnF | 601 | 758900 | 3,95 | 29988 |
| MDV27 | *Dendroctonus ponderosae* | COI | 220 | 723572 | 0,00 | 13 |
| MDV27 | *Salix sp.* | 28S | 503 | 723572 | 8,42 | 60924 |
| MDV27 | *Carex sp.* | rbcL | 318 | 723572 | 0,94 | 6772 |
| MDV27 | *Phragmites australis* | trnL - trnF | 1041 | 723572 | 1,84 | 13314 |
| MDV27 | *Phragmites australis* | matK | 890 | 723572 | 0,38 | 2748 |
| MDV27 | *Phragmites australis* | rbcL | 630 | 723572 | 7,89 | 57115 |
| MDV27 | *Deroceras laeve* | COI | 211 | 723572 | 0,00 | 14 |
| MDV27 | *Limnephilus flavicornis* | COI | 414 | 723572 | 0,01 | 38 |
| MDV29 | *Baetis rhodani* | COI | 706 | 764060 | 0,04 | 319 |
| MDV29 | *Betula pubescens* | matK | 889 | 764060 | 0,11 | 845 |
| MDV29 | *Asellus aquaticus* | COI | 365 | 764060 | 0,01 | 56 |
| MDV29 | *Populus nigra* | matK | 957 | 764060 | 0,01 | 82 |
| MDV29 | *Brachytron pratense* | COI | 709 | 764060 | 8,62 | 65895 |
| MDV29 | *Carex sp.* | trnL - trnF | 392 | 764060 | 0,00 | 33 |
| MDV29 | *Phragmites australis* | trnL - trnF | 1062 | 764060 | 2,58 | 19678 |
| MDV29 | *Phragmites australis* | matK | 892 | 764060 | 0,24 | 1817 |
| MDV29 | *Pleurochaete squarrosa* | trnL - trnF | 502 | 764060 | 2,49 | 19049 |
| MDV30 | *Pericoma blandula* | COI | 713 | 827212 | 4,89 | 40467 |
| MDV30 | *Alnus glutinosa* | trnL - trnF | 1055 | 827212 | 4,38 | 36269 |
| MDV30 | *Ilyocoris cimicoides* | COI | 352 | 827212 | 0,04 | 332 |
| MDV30 | *Salix sp.* | 28S | 241 | 827212 | 0,00 | 9 |
| MDV30 | *Juncus effusus* | 28S | 250 | 827212 | 1,72 | 14205 |
| MDV30 | *Phragmites australis* | matK | 889 | 827212 | 0,25 | 2035 |
| MDV30 | *Phragmites australis* | trnL - trnF | 520 | 827212 | 0,39 | 3240 |
| MDV30 | *Chionoloma tenuirostre* | COI | 624 | 827212 | 1,66 | 13692 |
| MDV30 | *Pleurochaete squarrosa* | trnL - trnF | 521 | 827212 | 4,37 | 36173 |
| MDV32 | *Gammarus pulex* | COI | 709 | 722194 | 0,05 | 372 |
| MDV32 | *Cecidomyiidae sp.* | COI | 365 | 722194 | 0,01 | 39 |
| MDV32 | *Alnus glutinosa* | matK | 455 | 722194 | 0,01 | 50 |
| MDV32 | *Alnus glutinosa* | trnL - trnF | 343 | 722194 | 0,01 | 66 |
| MDV32 | *Quercus sp.* | rbcL | 343 | 722194 | 1,75 | 12638 |
| MDV32 | *Hemiptera sp.* | COI | 233 | 722194 | 0,02 | 133 |
| MDV32 | *Populus nigra* | trnL - trnF | 1042 | 722194 | 0,28 | 2003 |
| MDV32 | *Populus nigra* | matK | 987 | 722194 | 0,46 | 3356 |
| MDV32 | *Phragmites australis* | trnL - trnF | 991 | 722194 | 4,690746 | 33876 |
| MDV32 | *Phragmites australis* | matK | 973 | 722194 | 0,65 | 4708 |
| MDV32 | *Phragmites australis* | rbcL | 582 | 722194 | 7,98 | 57654 |
| MDV32 | *Pleurochaete squarrosa* | trnL - trnF | 312 | 722194 | 0,24 | 1721 |
| MDV33 | *Nymphaea alba* | rbcL | 676 | 662592 | 96,64 | 640352 |
| MDV34 | *Ilyocoris cimicoides* | COI | 364 | 752330 | 1,54 | 11554 |
| MDV34 | *Notonecta glauca* | COI | 214 | 752330 | 0,00 | 16 |
| MDV34 | *Carex sp.* | rbcL | 425 | 752330 | 5,95 | 44741 |
| MDV34 | *Juncus effusus* | 28S | 345 | 752330 | 7,34 | 55233 |
| MDV34 | *Juncus effusus* | 28S | 272 | 752330 | 6,52 | 49054 |
| MDV34 | *Phragmites australis* | trnL - trnF | 1049 | 752330 | 8,44 | 63533 |
| MDV34 | *Phragmites australis* | rbcL | 542 | 752330 | 9,92 | 74616 |
| MDV35 | *Alnus alnobetula* | 28S | 523 | 677104 | 12,07 | 81749 |
| MDV35 | *Betula pubescens* | rbcL | 971 | 677104 | 11,69 | 79132 |
| MDV35 | *Betula pubescens* | rbcL | 683 | 677104 | 10,21 | 69105 |
| MDV35 | *Parapoynx stratiotata* | COI | 269 | 677104 | 0,00 | 21 |
| MDV35 | *Phragmites australis* | trnL - trnF | 1026 | 677104 | 0,07 | 468 |
| MDV35 | *Phragmites australis* | matK | 890 | 677104 | 0,15 | 1006 |
| MDV35 | *Phragmites australis* | rbcL | 640 | 677104 | 9,96 | 67412 |
| MDV35 | *Pleurochaete squarrosa* | trnL - trnF | 318 | 677104 | 4,62 | 31300 |
| MDV35 | *Athripsodes aterrimus* | COI | 216 | 677104 | 0,00 | 17 |
| MDV36 | *Salix sp.* | rbcL | 276 | 625446 | 0,00 | 13 |
| MDV36 | *Nymphaea alba* | rbcL | 598 | 625446 | 9,08 | 56813 |
| MDV36 | *Phragmites australis* | matK | 213 | 625446 | 0,00 | 13 |
| MDV36 | *Phragmites australis* | rbcL | 637 | 625446 | 8,66 | 54152 |
| MDV36 | *Phragmites australis* | trnL - trnF | 1040 | 625446 | 0,05 | 338 |

**Figure S6.** Faecal samples (N = 32) from European pond turtle (*Emys orbicularis*) analyzed using our long metabarcoding approach and *de novo* assembly. Sample and turtle identities, along with sex (M = Male, F = female and U = Undetermined), maturity (A = Adult, J = Juvenile) of each individual as well as genus/species of each prey DNA fragment sequenced and identified have been included. The reference alignment length, corresponding to the number of bp of each contig (amplicon assembly), and respective identity matches have been given using the NCBI nucleotide database BLAST search.

| **Turtle_ID** | **Sex** | **Maturity** | **Sample_ID** | **Class** | **Order** | **Species** | **% identity match** | **Reference alignment length (in bp)** |
| --- | --- | --- | --- | --- | --- | --- | --- | --- |
| 141 | M | A | MDV01 | Amphibia | Anura | *Bufo bufo* | 99.4 | 217 |
| 141 | M | A | MDV01 | Insecta | Diptera | *Endochironomus tendens* | 98.7 | 706 |
| 141 | M | A | MDV01 | Insecta | Trichoptera | *Limnephilus flavicornis* | 100.0 | 709 |
| 141 | M | A | MDV01 | Insecta | Hemiptera | *Ovatus crataegarius* | 100.0 | 239 |
| 141 | M | A | MDV01 | Insecta | Lepidoptera | *Phaenopsectra punctipes* | 97.0 | 233 |
| 141 | M | A | MDV01 | Liliopsida | Poales | *Phragmites australis* | 100.0 | 1041 |
| 141 | M | A | MDV01 | Bryopsida | Pottiales | *Streblotrichum convolutum* | 99.2 | 650 |
| 437 | U | J | MDV02 | Magnoliopsida | Fagales | *Alnus glutinosa* | 99.7 | 1055 |
| 437 | U | J | MDV02 | Magnoliopsida | Fagales | *Betula pubescens* | 98.3 | 834 |
| 437 | U | J | MDV02 | Liliopsida | Poales | *Carex sp.* | 99.8 | 438 |
| 437 | U | J | MDV02 | Liliopsida | Poales | *Juncus effusus* | 99.3 | 272 |
| 437 | U | J | MDV02 | Liliopsida | Poales | *Phragmites australis* | 100.0 | 1041 |
| 437 | U | J | MDV02 | Magnoliopsida | Malpighiales | *Populus alba* | 100.0 | 584 |
| 437 | U | J | MDV02 | Magnoliopsida | Malpighiales | *Populus nigra* | 98.6 | 278 |
| 437 | F | A | MDV03 | Magnoliopsida | Fagales | *Alnus alnobetula* | 98.9 | 548 |
| 437 | F | A | MDV03 | Malacostrca | Isopoda | *Asellus aquaticus* | 98.2 | 365 |
| 437 | F | A | MDV03 | Insecta | Odonata | *Brachytron pratense* | 98.3 | 385 |
| 437 | F | A | MDV03 | Liliopsida | Poales | *Carex sp.* | 100.0 | 318 |
| 437 | F | A | MDV03 | Mammalia | Rodentia | *Castor fiber* | 98.1 | 259 |
| 437 | F | A | MDV03 | Insecta | Hemiptera | *Ilyocoris cimicoides* | 98.8 | 366 |
| 437 | F | A | MDV03 | Liliopsida | Poales | *Juncus effusus* | 99.3 | 285 |
| 437 | F | A | MDV03 | Insecta | Trichoptera | *Limnephilus flavicornis* | 100.0 | 709 |
| 437 | F | A | MDV03 | Liliopsida | Poales | *Phragmites australis* | 100.0 | 257 |
| 437 | F | A | MDV03 | Magnoliopsida | Malpighiales | *Populus alba* | 99.7 | 307 |
| 437 | F | A | MDV03 | Insecta | Odonata | *Pyrrhosoma nymphula* | 99.8 | 706 |
| 212 | M | A | MDV04 | Magnoliopsida | Fagales | *Alnus alnobetula* | 98.9 | 472 |
| 212 | M | A | MDV04 | Magnoliopsida | Fagales | *Betula pubescens* | 99.9 | 889 |
| 212 | M | A | MDV04 | Amphibia | Anura | *Bufo bufo* | 98.8 | 328 |
| 212 | M | A | MDV04 | Insecta | Hemiptera | *Ilyocoris cimicoides* | 99.8 | 727 |
| 212 | M | A | MDV04 | Insecta | Trichoptera | *Limnephilus flavicornis* | 99.8 | 707 |
| 212 | M | A | MDV04 | Liliopsida | Poales | *Phragmites australis* | 99.8 | 1040 |
| 212 | M | A | MDV04 | Liliopsida | Alismatales | *Potamogeton perfoliatus* | 99.7 | 1056 |
| 212 | M | A | MDV04 | Magnoliopsida | Fagales | *Quercus sp.* | 99.3 | 889 |
| 212 | M | A | MDV04 | Magnoliopsida | Malpighiales | *Salix sp.* | 99.7 | 594 |
| 431 | M | A | MDV05 | Insecta | Odonata | *Anax imperator* | 99.4 | 365 |
| 431 | M | A | MDV05 | Magnoliopsida | Fagales | *Betula pubescens* | 99.7 | 587 |
| 431 | M | A | MDV05 | Insecta | Ephemeroptera | *Caenis horaria* | 98.0 | 233 |
| 431 | M | A | MDV05 | Insecta | Hemiptera | *Ilyocoris cimicoides* | 99.1 | 365 |
| 431 | M | A | MDV05 | Insecta | Trichoptera | *Limnephilus flavicornis* | 100.0 | 709 |
| 431 | M | A | MDV05 | Liliopsida | Poales | *Phragmites australis* | 100.0 | 1379 |
| 431 | M | A | MDV05 | Magnoliopsida | Malpighiales | *Populus alba* | 99.5 | 987 |
| 95 | M | A | MDV06 | Equisetopsida | Fagales | *Alnus alnobetula* | 98.9 | 548 |
| 95 | M | A | MDV06 | Magnoliopsida | Fagales | *Betula pubescens* | 99.8 | 901 |
| 95 | M | A | MDV06 | Liliopsida | Poales | *Carex sp.* | 100.0 | 426 |
| 95 | M | A | MDV06 | Insecta | Hemiptera | *Ilyocoris cimicoides* | 100.0 | 366 |
| 95 | M | A | MDV06 | Insecta | Trichoptera | *Limnephilus vittatus* | 99.2 | 316 |
| 95 | M | A | MDV06 | Liliopsida | Poales | *Phragmites australis* | 100.0 | 1041 |
| 95 | M | A | MDV06 | Magnoliopsida | Malpighiales | *Populus nigra* | 100.0 | 256 |
| 198 | F | A | MDV07 | Magnoliopsida | Fagales | *Alnus subcordata* | 98.3 | 176 |
| 198 | F | A | MDV07 | Liliopsida | Poales | *Carex sp.* | 96.5 | 541 |
| 198 | F | A | MDV07 | Bryopsida | Hypnales | *Hypnum cupressiforme* | 99.7 | 599 |
| 198 | F | A | MDV07 | Liliopsida | Poales | *Phragmites australis* | 100.0 | 1041 |
| 198 | F | A | MDV07 | Magnoliopsida | Malpighiales | *Populus alba* | 98.9 | 370 |
| 198 | F | A | MDV07 | Magnoliopsida | Malpighiales | *Populus nigra* | 99.8 | 455 |
| 297 | M | A | MDV08 | Magnoliopsida | Fagales | *Alnus alnobetula* | 98.3 | 696 |
| 297 | M | A | MDV08 | Malacostrca | Isopoda | *Asellus aquaticus* | 98.4 | 387 |
| 297 | M | A | MDV08 | Bryopsida | Pottiales | *Barbula unguiculata* | 98.1 | 579 |
| 297 | M | A | MDV08 | Liliopsida | Poales | *Carex sp.* | 100.0 | 398 |
| 297 | M | A | MDV08 | Insecta | Diptera | *Chironomus pallidivittatus* | 99.0 | 423 |
| 297 | M | A | MDV08 | Insecta | Diptera | *Endochironomus tendens* | 98.1 | 706 |
| 297 | M | A | MDV08 | Malacostrca | Amphipoda | *Gammarus fossarum* | 97.2 | 365 |
| 297 | M | A | MDV08 | Insecta | Trichoptera | *Limnephilus flavicornis* | 100.0 | 706 |
| 297 | M | A | MDV08 | Liliopsida | Poales | *Phragmites australis* | 100.0 | 1040 |
| 297 | M | A | MDV08 | Malacostrca | Isopoda | *Proasellus coxalis* | 99.2 | 387 |
| 75 | M | A | MDV09 | Magnoliopsida | Fagales | *Alnus alnobetula* | 99.1 | 579 |
| 75 | M | A | MDV09 | Amphibia | Anura | *Bufo bufo* | 98.0 | 207 |
| 75 | M | A | MDV09 | Liliopsida | Poales | *Carex sp.* | 100.0 | 379 |
| 75 | M | A | MDV09 | Liliopsida | Poales | *Phragmites australis* | 99.7 | 891 |
| 75 | M | A | MDV09 | Bryopsida | Pottiales | *Pleurochaete squarrosa* | 99.6 | 504 |
| 75 | M | A | MDV09 | Magnoliopsida | Malpighiales | *Populus alba* | 99.3 | 284 |
| 75 | M | A | MDV09 | Bryopsida | Pottiales | *Pottiopsis caespitosa* | 98.7 | 620 |
| 75 | M | A | MDV09 | Magnoliopsida | Fagales | *Quercus sp.* | 99.1 | 915 |
| 75 | M | A | MDV09 | Bryopsida | Pottiales | *Tortella tortuosa* | 99.6 | 504 |
| 123 | F | A | MDV10 | Magnoliopsida | Fagales | *Alnus glutinosa* | 100.0 | 265 |
| 123 | F | A | MDV10 | Magnoliopsida | Fagales | *Betula pubescens* | 99.4 | 889 |
| 123 | F | A | MDV10 | Insecta | Ephemeroptera | *Cloeon dipterum* | 99.8 | 706 |
| 123 | F | A | MDV10 | Insecta | Lepidoptera | *Pheosia tremula* | 98.9 | 212 |
| 123 | F | A | MDV10 | Gastropoda | Hygrophila | *Radix auricularia* | 99.2 | 263 |
| 123 | F | A | MDV10 | Magnoliopsida | Malpighiales | *Salix sp.* | 100.0 | 282 |
| 438 | U | J | MDV11 | Magnoliopsida | Fagales | *Alnus glutinosa* | 99.8 | 961 |
| 438 | U | J | MDV11 | Insecta | Odonata | *Calopteryx virgo* | 98.9 | 712 |
| 438 | U | J | MDV11 | Liliopsida | Poales | *Carex sp.* | 95.9 | 591 |
| 438 | U | J | MDV11 | Insecta | Odonata | *Coenagrion pulchellum* | 99.8 | 710 |
| 438 | U | J | MDV11 | Polypodiopsida | Equisetales | *Equisetum hyemale* | 100.0 | 275 |
| 438 | U | J | MDV11 | Insecta | Hemiptera | *Ilyocoris cimicoides* | 99.8 | 709 |
| 438 | U | J | MDV11 | Insecta | Trichoptera | *Limnephilus flavicornis* | 100.0 | 712 |
| 438 | U | J | MDV11 | Liliopsida | Poales | *Phragmites australis* | 99.9 | 1041 |
| 438 | U | J | MDV11 | Bryopsida | Pottiales | *Pleurochaete squarrosa* | 99.8 | 502 |
| 438 | U | J | MDV11 | Magnoliopsida | Malpighiales | *Salix sp.* | 99.1 | 338 |
| 438 | U | J | MDV11 | Bryopsida | Pottiales | *Tortella tortuosa* | 98.1 | 635 |
| 131 | M | A | MDV12 | Magnoliopsida | Fagales | *Alnus glutinosa* | 98.1 | 421 |
| 131 | M | A | MDV12 | Magnoliopsida | Fagales | *Betula pubescens* | 100.0 | 497 |
| 131 | M | A | MDV12 | Gastropoda | Littorinimorpha | *Bithynia tentaculata* | 98.5 | 364 |
| 131 | M | A | MDV12 | Liliopsida | Poales | *Carex sp.* | 99.6 | 269 |
| 131 | M | A | MDV12 | Actinoptergii | Cypriniformes | *Cyprinus carpio* | 99.7 | 300 |
| 131 | M | A | MDV12 | Insecta | Trichoptera | *Limnephilus flavicornis* | 99.7 | 436 |
| 131 | M | A | MDV12 | Insecta | Hemiptera | *Notonecta glauca* | 98.1 | 266 |
| 131 | M | A | MDV12 | Liliopsida | Poales | *Phragmites australis* | 100.0 | 1050 |
| 131 | M | A | MDV12 | Bryopsida | Pottiales | *Pleurochaete squarrosa* | 99.6 | 502 |
| 131 | M | A | MDV12 | Bryopsida | Pottiales | *Tortella tortuosa* | 98.0 | 669 |
| 400 | M | A | MDV14 | Liliopsida | Poales | *Carex sp.* | 99.3 | 282 |
| 400 | M | A | MDV14 | Liliopsida | Poales | *Cladium mariscus* | 100.0 | 870 |
| 400 | M | A | MDV14 | Insecta | Diptera | *Cricotopus bicinctus* | 98.3 | 229 |
| 400 | M | A | MDV14 | Liliopsida | Poales | *Juncus effusus* | 99.3 | 282 |
| 400 | M | A | MDV14 | Liliopsida | Poales | *Phragmites australis* | 100.0 | 975 |
| 400 | M | A | MDV14 | Magnoliopsida | Fagales | *Quercus sp.* | 99.8 | 543 |
| 400 | M | A | MDV14 | Insecta | Odonata | *Sympetrum striolatum* | 98.8 | 364 |
| U | M | A | MDV15 | Magnoliopsida | Fagales | *Alnus alnobetula* | 98.9 | 474 |
| U | M | A | MDV15 | Amphibia | Anura | *Bufo bufo* | 99.1 | 708 |
| U | M | A | MDV15 | Insecta | Trichoptera | *Limnephilus flavicornis* | 99.6 | 271 |
| U | M | A | MDV15 | Liliopsida | Poales | *Phragmites australis* | 100.0 | 1041 |
| U | M | A | MDV15 | Magnoliopsida | Fagales | *Quercus sp.* | 99.7 | 601 |
| 322 | F | A | MDV16 | Insecta | Trichoptera | *Limnephilus flavicornis* | 98.8 | 709 |
| 322 | F | A | MDV16 | Liliopsida | Poales | *Phragmites australis* | 100.0 | 1041 |
| 322 | F | A | MDV16 | Magnoliopsida | Malpighiales | *Populus alba* | 100.0 | 168 |
| 322 | F | A | MDV16 | Magnoliopsida | Malpighiales | *Salix sp.* | 99.4 | 347 |
| 441 | U | J | MDV17 | Malacostrca | Isopoda | *Asellus aquaticus* | 98.5 | 365 |
| 441 | U | J | MDV17 | Magnoliopsida | Fagales | *Betula pubescens* | 99.9 | 823 |
| 441 | U | J | MDV17 | Liliopsida | Poales | *Carex sp.* | 100.0 | 232 |
| 441 | U | J | MDV17 | Polypodiopsida | Equisetales | *Equisetum ramosissimum* | 99.1 | 234 |
| 441 | U | J | MDV17 | Insecta | Trichoptera | *Limnephilus flavicornis* | 100.0 | 709 |
| 441 | U | J | MDV17 | Insecta | Diptera | *Cricotopus triannulatus* | 100.0 | 709 |
| 441 | U | J | MDV17 | Liliopsida | Poales | *Phragmites australis* | 100.0 | 1040 |
| 441 | U | J | MDV17 | Magnoliopsida | Fagales | *Quercus sp.* | 99.7 | 922 |
| 441 | U | J | MDV17 | Magnoliopsida | Malpighiales | *Salix sp.* | 98.4 | 306 |
| 170 | F | A | MDV18 | Insecta | Coleoptera | *Agrilus angustulus* | 100.0 | 179 |
| 170 | F | A | MDV18 | Magnoliopsida | Fagales | *Alnus incana* | 99.8 | 493 |
| 170 | F | A | MDV18 | Gastropoda | Littorinimorpha | *Bithynia tentaculata* | 97.5 | 281 |
| 170 | F | A | MDV18 | Liliopsida | Poales | *Carex sp.* | 99.2 | 255 |
| 170 | F | A | MDV18 | Liliopsida | Poales | *Cladium sp.* | 100.0 | 868 |
| 170 | F | A | MDV18 | Insecta | Hemiptera | *Ilyocoris cimicoides* | 99.1 | 365 |
| 170 | F | A | MDV18 | Liliopsida | Poales | *Phragmites australis* | 100.0 | 975 |
| 170 | F | A | MDV18 | Liliopsida | Alismatales | *Potamogeton perfoliatus* | 99.7 | 984 |
| 170 | F | A | MDV18 | Magnoliopsida | Malpighiales | *Salix sp.* | 100.0 | 241 |
| 170 | F | A | MDV18 | Bryopsida | Pottiales | *Tortella tortuosa* | 98.9 | 560 |
| 309 | F | A | MDV19 | Insecta | Trichoptera | *Limnephilus flavicornis* | 99.8 | 709 |
| 309 | F | A | MDV19 | Liliopsida | Poales | *Phragmites australis* | 100.0 | 1042 |
| 1 | F | A | MDV21 | Magnoliopsida | Fagales | *Alnus incana* | 99.3 | 579 |
| 1 | F | A | MDV21 | Malacostrca | Isopoda | *Armadillidium nasatum* | 99.1 | 224 |
| 1 | F | A | MDV21 | Liliopsida | Poales | *Phragmites australis* | 99.7 | 385 |
| 1 | F | A | MDV21 | Magnoliopsida | Malpighiales | *Populus alba* | 100.0 | 262 |
| 105 | M | A | MDV22 | Magnoliopsida | Fagales | *Betula sp.* | 99.5 | 634 |
| 105 | M | A | MDV22 | Amphibia | Anura | *Bufo bufo* | 98.0 | 208 |
| 105 | M | A | MDV22 | Insecta | Coleoptera | *Donacia clavipes* | 97.3 | 213 |
| 105 | M | A | MDV22 | Malacostrca | Amphipoda | *Gammarus fossarum* | 98.5 | 263 |
| 105 | M | A | MDV22 | Bryopsida | Hypnales | *Hypnum cupressiforme* | 100.0 | 249 |
| 105 | M | A | MDV22 | Liliopsida | Poales | *Phragmites australis* | 100.0 | 1041 |
| 105 | M | A | MDV22 | Magnoliopsida | Malpighiales | *Populus alba* | 99.9 | 744 |
| 69 | F | A | MDV23 | Magnoliopsida | Fagales | *Alnus alnobetula* | 99.1 | 579 |
| 69 | F | A | MDV23 | Magnoliopsida | Fagales | *Betula pubescens* | 100.0 | 308 |
| 69 | F | A | MDV23 | Gastropoda | Littorinimorpha | *Bithynia tentaculata* | 99.7 | 709 |
| 69 | F | A | MDV23 | Insecta | Ephemeroptera | *Caenis horaria* | 98.1 | 288 |
| 69 | F | A | MDV23 | Liliopsida | Poales | *Carex sp.* | 100.0 | 398 |
| 69 | F | A | MDV23 | Insecta | Odonata | *Coenagrion pulchellum* | 99.8 | 715 |
| 69 | F | A | MDV23 | Insecta | Trichoptera | *Limnephilus flavicornis* | 98.5 | 365 |
| 69 | F | A | MDV23 | Liliopsida | Poales | *Phragmites australis* | 99.8 | 547 |
| 69 | F | A | MDV23 | Bryopsida | Pottiales | *Pleurochaete squarrosa* | 98.5 | 274 |
| 69 | F | A | MDV23 | Liliopsida | Alismatales | *Potamogeton perfoliatus* | 99.6 | 528 |
| 69 | F | A | MDV23 | Bryopsida | Pottiales | *Tortella tortuosa* | 98.5 | 274 |
| 442 | M | A | MDV24 | Magnoliopsida | Fagales | *Alnus alnobetula* | 99.3 | 729 |
| 442 | M | A | MDV24 | Magnoliopsida | Fagales | *Betula pubescens* | 100.0 | 307 |
| 442 | M | A | MDV24 | Gastropoda | Littorinimorpha | *Bithynia tentaculata* | 97.2 | 365 |
| 442 | M | A | MDV24 | Liliopsida | Poales | *Carex sp.* | 100.0 | 398 |
| 442 | M | A | MDV24 | Liliopsida | Poales | *Phragmites australis* | 99.7 | 639 |
| 442 | M | A | MDV24 | Pinopsida | Pinales | *Picea sp.* | 99.5 | 601 |
| 442 | M | A | MDV24 | Magnoliopsida | Malpighiales | *Populus alba* | 99.6 | 637 |
| 442 | M | A | MDV24 | Magnoliopsida | Fagales | *Quercus sp.* | 100.0 | 344 |
| 267 | M | A | MDV25 | Insecta | Trichoptera | *Limnephilus flavicornis* | 100.0 | 622 |
| 267 | M | A | MDV25 | Liliopsida | Poales | *Phragmites australis* | 100.0 | 1040 |
| 60 | M | A | MDV26 | Liliopsida | Poales | *Carex sp.* | 100.0 | 398 |
| 60 | M | A | MDV26 | Liliopsida | Poales | *Phragmites australis* | 100.0 | 1041 |
| 60 | M | A | MDV26 | Bryopsida | Pottiales | *Pleurochaete squarrosa* | 99.6 | 503 |
| 60 | M | A | MDV26 | Magnoliopsida | Malpighiales | *Populus alba* | 99.8 | 629 |
| 60 | M | A | MDV26 | Bryopsida | Pottiales | *Tortella tortuosa* | 99.3 | 601 |
| 216 | M | A | MDV27 | Liliopsida | Poales | *Carex sp.* | 100.0 | 318 |
| 216 | M | A | MDV27 | Insecta | Coleoptera | *Dendroctonus ponderosae* | 99.5 | 220 |
| 216 | M | A | MDV27 | Gastropoda | Stylommatophora | *Deroceras laeve* | 99.1 | 211 |
| 216 | M | A | MDV27 | Insecta | Trichoptera | *Limnephilus flavicornis* | 98.5 | 414 |
| 216 | M | A | MDV27 | Malacostrca | Isopoda | *Philoscia muscorum* | 99.1 | 211 |
| 216 | M | A | MDV27 | Liliopsida | Poales | *Phragmites australis* | 100.0 | 1041 |
| 216 | M | A | MDV27 | Pinopsida | Pinales | *Picea sp.* | 100.0 | 370 |
| 216 | M | A | MDV27 | Magnoliopsida | Fagales | *Quercus sp.* | 99.8 | 415 |
| 216 | M | A | MDV27 | Magnoliopsida | Malpighiales | *Salix sp.* | 99.8 | 503 |
| 157 | M | A | MDV29 | Malacostrca | Isopoda | *Asellus aquaticus* | 97.5 | 365 |
| 157 | M | A | MDV29 | Insecta | Ephemeroptera | *Baetis rhodani* | 99.5 | 706 |
| 157 | M | A | MDV29 | Magnoliopsida | Fagales | *Betula pubescens* | 99.6 | 889 |
| 157 | M | A | MDV29 | Insecta | Odonata | *Brachytron pratense* | 100.0 | 709 |
| 157 | M | A | MDV29 | Liliopsida | Poales | *Carex sp.* | 99.2 | 392 |
| 157 | M | A | MDV29 | Liliopsida | Poales | *Phragmites australis* | 100.0 | 1062 |
| 157 | M | A | MDV29 | Bryopsida | Pottiales | *Pleurochaete squarrosa* | 99.6 | 502 |
| 157 | M | A | MDV29 | Magnoliopsida | Malpighiales | *Populus nigra* | 99.5 | 957 |
| 157 | M | A | MDV29 | Magnoliopsida | Fagales | *Quercus sp.* | 97.8 | 503 |
| 166 | M | A | MDV30 | Magnoliopsida | Fagales | *Alnus glutinosa* | 99.9 | 1055 |
| 166 | M | A | MDV30 | Liliopsida | Poales | *Carex sp.* | 99.2 | 250 |
| 166 | M | A | MDV30 | Bryopsida | Pottiales | *Chionoloma tenuirostre* | 99.2 | 624 |
| 166 | M | A | MDV30 | Insecta | Hemiptera | *Ilyocoris cimicoides* | 99.7 | 352 |
| 166 | M | A | MDV30 | Liliopsida | Poales | *Juncus effusus* | 99.2 | 250 |
| 166 | M | A | MDV30 | Insecta | Diptera | *Pericoma blandula* | 98.2 | 713 |
| 166 | M | A | MDV30 | Liliopsida | Poales | *Phragmites australis* | 99.7 | 889 |
| 166 | M | A | MDV30 | Pinopsida | Pinales | *Picea sp.* | 99.8 | 998 |
| 166 | M | A | MDV30 | Bryopsida | Pottiales | *Pleurochaete squarrosa* | 100.0 | 521 |
| 166 | M | A | MDV30 | Magnoliopsida | Malpighiales | *Salix sp.* | 99.2 | 241 |
| 92 | M | A | MDV32 | Magnoliopsida | Fagales | *Alnus glutinosa* | 99.3 | 455 |
| 92 | M | A | MDV32 | Insecta | Diptera | *Cecidomyiidae sp.* | 99.4 | 365 |
| 92 | M | A | MDV32 | Malacostrca | Amphipoda | *Gammarus pulex* | 99.8 | 709 |
| 92 | M | A | MDV32 | Insecta | Hemiptera | *Hemiptera sp.* | 100.0 | 233 |
| 92 | M | A | MDV32 | Liliopsida | Poales | *Phragmites australis* | 100.0 | 991 |
| 92 | M | A | MDV32 | Bryopsida | Pottiales | *Pleurochaete squarrosa* | 100.0 | 312 |
| 92 | M | A | MDV32 | Magnoliopsida | Malpighiales | *Populus alba* | 100.0 | 340 |
| 92 | M | A | MDV32 | Magnoliopsida | Malpighiales | *Populus nigra* | 99.6 | 1042 |
| 92 | M | A | MDV32 | Magnoliopsida | Fagales | *Quercus sp.* | 100.0 | 343 |
| 415 | M | A | MDV33 | Magnoliopsida | Nymphaeales | *Nymphaea alba* | 99.2 | 676 |
| U | U | J | MDV34 | Liliopsida | Poales | *Carex sp.* | 99.8 | 425 |
| U | U | J | MDV34 | Insecta | Hemiptera | *Ilyocoris cimicoides* | 98.2 | 364 |
| U | U | J | MDV34 | Liliopsida | Poales | *Juncus effusus* | 99.3 | 272 |
| U | U | J | MDV34 | Insecta | Hemiptera | *Notonecta glauca* | 99.5 | 214 |
| U | U | J | MDV34 | Liliopsida | Poales | *Phragmites australis* | 100.0 | 1049 |
| 306 | M | A | MDV35 | Magnoliopsida | Fagales | *Alnus alnobetula* | 99.0 | 523 |
| 306 | M | A | MDV35 | Insecta | Trichoptera | *Athripsodes aterrimus* | 97.7 | 216 |
| 306 | M | A | MDV35 | Magnoliopsida | Fagales | *Betula pubescens* | 99.9 | 971 |
| 306 | M | A | MDV35 | Insecta | Lepidoptera | *Parapoynx stratiotata* | 96.2 | 269 |
| 306 | M | A | MDV35 | Liliopsida | Poales | *Phragmites australis* | 99.3 | 1026 |
| 306 | M | A | MDV35 | Bryopsida | Pottiales | *Pleurochaete squarrosa* | 99.4 | 315 |
| 306 | M | A | MDV35 | Magnoliopsida | Fagales | *Quercus sp.* | 99.4 | 314 |
| 306 | M | A | MDV35 | Bryopsida | Pottiales | *Tortella tortuosa* | 100.0 | 243 |
| 436 | U | J | MDV36 | Liliopsida | Poales | *Phragmites australis* | 100.0 | 1040 |
| 436 | U | J | MDV36 | Magnoliopsida | Malpighiales | *Salix sp.* | 100.0 | 276 |
